# Selenium‐binding protein 1 inhibits malignant progression and induces apoptosis via distinct mechanisms in non‐small cell lung cancer

**DOI:** 10.1002/cam4.6309

**Published:** 2023-08-22

**Authors:** Ying Zhu, Qiang Pu, Qiongyin Zhang, Yang Liu, Yongfang Ma, Yue Yuan, Lunxu Liu, Wen Zhu

**Affiliations:** ^1^ State Key Laboratory of Biotherapy and Cancer Center West China Hospital, Sichuan University Chengdu Sichuan China; ^2^ Department of Thoracic Surgery Institute of Thoracic Oncology, West China Hospital, Sichuan University Chengdu Sichuan China

**Keywords:** caspase‐3 pathway, GPX1, NSCLC, PI3K/AKT/mTOR pathway, SELENBP1, tumor suppressor

## Abstract

**Background:**

Selenium is an essential trace element in the human body. In epidemiological and clinical studies, Se supplementation significantly reduced the incidence of lung cancer in individuals with low baseline Se levels. The significant action of selenium is based on the selenium‐containing protein as a mediator. Of note, the previous studies reported that the expression of selenium‐binding protein 1 (SELENBP1) was obviously decreased in many human cancer tissues including non‐small cell lung cancer (NSCLC). However, its roles in the origin and development of NSCLC are still unclear.

**Methods:**

The expression of SELENBP1 was measured by qRT‐PCR, Western blotting and IHC in our collected clinical NSCLC tissues and cell lines. Next, the CCK‐8, colony formation, wound‐haeling, Millicell, Transwell, FCM assay, and in vivo xenograft model were performed to explore the function of SELENBP1 in NSCLC. The molecular mechanisms of SELENBP1 were investigated by Western blotting or IF assay.

**Results:**

We further identified that the expression of SELENBP1 was significantly decreased in NSCLC tissues in TCGA database and 45 out of 59 collected clinical NSCLC tissues compared with adjacent nontumor tissues, as well as in four NSCLC cell lines compared with normal lung cells. Particularly, we unexpectedly discovered that SELENBP1 was obviously expressed in alveolar type 2 (AT‐II) cells for the first time. Then, a series of in vitro experiments uncovered that overexpression of SELENBP1 inhibited the proliferation, migration, and invasion of NSCLC cells, and induced cell apoptosis. Moreover, overexpression of SELENBP1 also inhibited growth and induced apoptosis of NSCLC cells in vivo. Mechanistically, we demonstrated that overexpression of SELENBP1 inhibited the malignant characteristics of NSCLC cells in part via inactivating the PI3K/AKT/mTOR signal pathway. Meanwhile, we found that overexpression of SELENBP1 inducing the apoptosis of NSCLC cells was associated with the activation of caspase‐3 signaling pathway under nonhigh level of oxidative stress, but overexpression of SELENBP1 facilitating the cell apoptosis might be related to its combining with GPX1 and colocalizing in the nucleus under high level of oxidative stress.

**Conclusions:**

Our findings highlighted that SELENBP1 was an important tumor suppressor during the origin and development of NSCLC. It may help to discover novel biomarkers or drug therapy targets for NSCLC.

## 1 INTRODUCTION

Lung cancer (LC) has long been regarded as the major cause of cancer‐related deaths worldwide, ranking second in incidence and first deaths of malignant tumors.^1^ In 2020, 2.2 million people were diagnosed with LC, and 1.8 million people died as a result of the disease.^1^ The 5‐year overall survival (OS) rate of patients with LC is around 15%,^2^, ^3^ which has remained unaltered despite advances in surgical techniques and molecular targeting therapies in the last several decades.^4^ Poor outcomes of LC are correlated with the propensity for metastasis and diagnosis at advanced stages.^4^, ^5^ Non‐small cell lung cancer (NSCLC) account for no <85% in all LC cases.^6^ So far, the understanding of potential molecular mechanisms for the origin and development of NSCLC is still limited, and there are no resultful biomarkers for early diagnosis of NSCLC. Therefore, a better understanding of potential mechanisms may help to discover the novel biomarkers or drug therapy targets for NSCLC.

Selenium (Se), discovered by Swedish doctor Jacob Berzelius in 1817,^7^ is an essential trace element in the human body. Se undergoes complex biochemical processes, and exists in cellular in the form of selenium‐containing protein.^8^, ^9^ The important role of Se is based on the selenium‐containing protein as a mediator.^10^ Human selenium‐containing proteins have three categories, the selenium‐binding proteins such as SELENBP1, the proteins containing selenocysteine such as GPX1 (glutathione peroxidase 1) and the sulfur‐containing proteins in which sulfur is incorrectly substituted by Se.^11^ Se is incorporated into selenium‐containing proteins that have diverse effects, such as antioxidant, immune‐endocrine effects, anti‐inflammatory functions, metabolic cycling, and cellular homeostasis.^12^, ^13^ Moreover, many epidemiologic studies showed that Se has cancer‐preventing effects.^14^, ^15^, ^16^ However, the antitumor mechanisms of selenium remain to be investigated. Resplendently, selenium‐binding protein 1 (SELENBP1) may function as a mediator of selenium's anticancer actions.^11^, ^17^ *SELENBP1*, located on chromosome 1 at q21‐22, encodes a highly conserved member of the selenium‐binding protein family.^18^ Human SELENBP1 is the homolog with a 56‐kD mouse selenium‐binding protein.^19^ Different from the proteins containing selenocysteine, Se is stably bound to SELENBP1.^11^, ^20^ SELENBP1 is ubiquitously expressed in nucleus and cytoplasm of many tissue cells including lung, kidney, liver, heart, as well as brain.^18^, ^21^ Previous studies found that the expression of SELENBP1 was dramatically downregulated in human cancer tissues including thyroid cancer, lung cancer, liver cancer, gastric cancer, kidney cancer and so on, and the downregulated expression of SELENBP1 was associated with the progression and poor prognosis of various cancers.^11^, ^17^ Besides, SELENBP1 was identified as a new human methanethiol oxidase (MTO), its mutations could lead to extra‐oral halitosis.^22^

Nowadays, more attentions have gradually focused on the roles and potential molecular mechanisms of SELENBP1 in tumorigenesis and development. In biological functions, many studies in vitro and in vivo have congruously reported that the overexpression of SELENBP1 notably suppressed the malignant characteristics of cancer cells in prostate cancer^23^ and colorectal cancer.^24^ Furthermore, the downregulated expression of SELENBP1 induced the migration and invasion of liver cancer cells SMMC7721.^25^ Mechanistically, the overexpression of SELENBP1 was uncovered to inhibit cell growth by decreasing the protein expression of hypoxia‐inducible factor‐1α (HIF‐1α) in prostate cancer cells,^23^ and remarkably restrained cell growth through activating p21 with p53‐independent way in bladder cancer cells.^26^ Besides, in prostate cells, SELENBP1 activated AMPK pathway to maintain prostate energy metabolism. But the downregulated expression of SELENBP1 was shown to restrain the activation of AMPK signaling and increased oxidative phosphorylation level, changed the energy metabolism pattern, and promoted the development of prostate cancer.^27^ However, a few studies reported that the expression of SELENBP1 was decreased in the most of NSCLC tissues compared with adjacent nontumor tissues, and the downregulated expression of SELENBP1 was correlated with poor outcomes.^28^, ^29^ Moreover, Deborah R et al.^30^ reported that SELENBP1 could inhibit the growth and metastasis of tumors in Kras^G12D^‐ driven mice lung adenocarcinoma model. Nevertheless, the biological functions and potential molecular mechanisms of SELENBP1 in human NSCLC cells have not yet been identified in any detail until now.

Therefore, in the present study, we sought to explore the effects of SELENBP1 in the origin and development of NSCLC in vitro and in vivo, and revealed the corresponding mechanisms of SELENBP1.

## 2 MATERIALS AND METHODS

### 2.1 Cell culture and established stable cell lines

The NSCLC cell lines A549, NCI‐H1299, NCI‐H358, SK‐MES‐1, and the normal lung epithelial cell line HBE were purchased from American Type Culture Collection (ATCC). The A549, NCI‐H1299, and NCI‐H358 cells were cultured in RPMI 1640 (Invitrogen), SK‐MES‐1 was cultured in Eagle's minimum Essential Medium (MEM; Invitrogen), and HBE cells were cultured in Dulbecco's Modified Eagle's Medium (DMEM; Invitrogen). All cells were maintained in medium containing 10% fetal bovine serum (FBS; Invitrogen/Gibco), 100 units of penicillin (Merck Millipore), and 100 μg of streptomycin (Merck Millipore) at 37°C incubator in 5% CO_2_.

Recombinant lentiviruses expressing SELENBP1 (Gene ID, 8991) and the control lentiviruses were purchased from HanBio. The stable SELENBP1‐overexpressing cell lines (A549‐SELENBP1 and H1299‐SELENBP1) and control cell lines (A549‐Control, H1299‐Control) were established through lentivirus infection, and puromycin (1 μg/mL; Merck Millipore) selection as previously described.^31^ The expression levels of SELENBP1 were measured by qRT‐PCR and western blotting.

### 2.2 Bioinformatics

The expression levels of SELENBP1 in NSCLC tissues (483 lung adenocarcinoma [LUAD] cases and 486 lung squamous cell carcinoma [LUSC] cases) and adjacent nontumor tissues from The Cancer Genomics Altas (TCGA) database were obtained from GEPIA: (GEPIA 2 [cancer‐pku.cn]), respectively. In addition, 240 LUAD cases and 242 LUSC cases were also detected by GEPIA: (GEPIA 2 [cancer‐pku.cn]) to preliminarily analyze the relationship between the expression of SELENBP1 and the overall survival (OS), respectively.

The levels of SELENBP1 in 515 LUAD cases and 492 LUSC cases in The Cancer Genomics Altas (TCGA) database were performed by the UALCAN: (http://ualcan.path.uab.edu/index.html) to preliminarily analyze the correlation between the expression of SELENBP1 and the disease stages of NSCLC, respectively.

### 2.3 Clinical specimens

In this study, 59 clinical NSCLC tissues and paired adjacent nontumor tissues were collected from the Department of Thoracic Surgery at West China Hospital. This study was performed in accordance with the Declaration of Helsinki. All the patients signed informed consent. Clinical information of the 59 NSCLC cases was presented in (Table S1).

### 2.4 Quantitative real‐time PCR (qRT‐PCR)

The total RNA of freshly prepared clinical tissues, and cells were extracted by using the TRIzol Reagent (Invitrogen) and then subjected to reverse transcription into the cDNA products by using Hiscript III qRT mix (Vazyme). qRT‐PCR process was conducted by using 2 × SYBR Green qPCR Master Mix (Yesen) on the Light Cycler 96 (Roche). qRT‐PCR primers are shown in (Table S2). The expression of target gene was analyzed by the 2^−ΔΔCt^ calculation method.

### 2.5 Hematoxylin and eosin (H&E) staining and immunohistochemical (IHC) staining

The clinical tissues and collected subcutaneous tumor tissues were fixed in fresh 4% paraformaldehyde at 4°C overnight, then dehydrated, paraffin‐embedded, and sectioned. The sections were deparaffinized, rehydrated, and subjected to H&E staining and IHC staining as described.^32^ In H&E staining, the tissues were stained with hematoxylin and eosin (Thermo Scientific). In IHC staining, the sections were deparaffinize, rehydrated, followed by antigen retrieval and immunostaining with different antibodies against SELENBP1 (1:200 dilution, Abcam, ab90135), SP‐C (1:200 dilution, Abcam, ab90716), Ki‐67 (1:200 dilution, Abcam, ab15580), PI3K (1:100 dilution, Abcam, ab151549), p‐PI3K (Tyr607; 1:200 dilution, Invitrogen, Catalog #PA5‐104853), AKT (1:200 dilution, CST, #4691), p‐AKT (Ser473; 1:100 dilution, CST, #4060), mTOR (1:100 dilution, CST, #2983), p‐mTOR (Ser2448; 1:200 dilution, Invitrogen, Catalog #44‐1125G), Caspase‐3 (1:200 dilution, Huabio, ET1602‐39), Cleaved‐Caspase‐3 (1:200 dilution, Affinity, AF7002), Bcl‐2 (1:200 dilution, Huabio, ET1603‐11), and Bax (1:200 dilution, Affinity, AF0120). Stains without primary antibody were used as negative control. Examined and recorded under a Zeiss Imager Z2 microscope (Carl Zeiss; 400×).

### 2.6 Western blotting

Western blotting was carried out as described previously.^31^ Primary antibodies against β‐actin (1:1000 dilution, Abcam, ab8226), SELENBP1 (1:1000 dilution, Abcam, ab90135), PI3K (1:1000 dilution, Abcam, ab151549), p‐PI3K (Tyr607; 1:1000 dilution, Invitrogen, Catalog #PA5‐104853), AKT (1:1000 dilution, CST, #4691), p‐AKT (Ser473; 1:2000 dilution, CST, #4060), mTOR (1:1000 dilution, CST, #2983), p‐mTOR (Ser2448; 1:1000 dilution, Invitrogen, Catalog #44‐1125G), Caspase‐3/p17 (1:1000 dilution, Proteintech, 66,470‐2‐Ig), Bcl‐2 (1:1000 dilution, Huabio, ET1603‐11), and Bax (1:1000 dilution, Affinity, AF0120) were used.

### 2.7 Cell proliferation assay and clone forming assay

The cells (A549‐Control, A549‐SELENBP1, H1299‐Control, and H1299‐SELENBP1) were seeded into 96‐well plates and each well contained around 5000 cells. Ten microliters of CCK‐8 solution (CCK‐8, Vazyme) was added to each well‐containing cells, and the cells were further cultured for 1 h at 37°C incubator. Cell plate was taken out, and the cell viability was measured at 450 nm using a microplate reader (Thermo Scientific).

The indicated cells were uniformly cultivated into 6‐well plates, each well has similar cells, and then, the cells were cultured for 10 days at 37°C incubator, fixed in 4% paraformaldehyde for 30 min, and washed with PBS; the indicated cells were stained by crystal violet stain (Sigma Diagnostics).

### 2.8 Wound‐healing assay

The cells (A549‐Control, A549‐SELENBP1, and H1299‐Control, H1299‐SELENBP1) were seeded into 6‐well plates, When the fusion of cells reached 95%, linear wounds were created by using a 200 μL pipette tip. Importantly, to remove cell proliferation to wound healing, both cells were cultured in serum‐free medium. To visualize wound healing, images were taken at 0, 24, and 48 h under a Zeiss Imager Z2 microscope (Carl Zeiss; 50×).

### 2.9 Cell migration assay

The cells (A549‐Control, A549‐SELENBP1 and H1299‐Control, H1299‐SELENBP1) were seeded into the Millicell chambers (8 μm pore size, Merck, Millipore) with serum‐free media and each Millicell chamber contained around 3.5 × 10^5^ cells. The chambers suspended on 24‐well plate. Media containing 10% FBS was added to the lower chamber, cultured at 37°C in the incubator for 24 h, and then fixed in 4% paraformaldehyde; the nonmigrating cells on the upper of the chambers were removed with cotton wool, and migratory cells located on the lower surface of the chamber were stained by crystal violet stain (Sigma Diagnostics). To count the migrated cells, pictures were taken using a Zeiss Imager Z2 microscope (Carl Zeiss; 200×).

### 2.10 Cell invasion assay

The transwell chambers (8‐μm pore size, Millipore) were coated with matrigel (BD Biosciences) diluted with RPMI‐1640 (1:5) on the upper chamber in advance. The cells (A549‐Control, A549‐SELENBP1, H1299‐Control, and H1299‐SELENBP1) were seeded into the transwell chambers (8‐μm pore size, Merck, Millipore) with serum‐free media and each transwell chamber contained around 4 × 10^5^ cells. Media containing 10% FBS was added to the lower chamber, cultured at 37°C incubator for 24 h, and then fixed in 4% paraformaldehyde; the noninvading cells on the upper of the chambers were removed with cotton wool; invasive cells located on the lower surface of the chamber were stained by crystal violet stain (Sigma Diagnostics). To count the invasive cells, pictures were taken using a Zeiss Imager Z2 microscope (Carl Zeiss; 200×).

### 2.11 Flow cytometry (FCM) assay

The indicated cells (A549‐Control, A549‐SELENBP1, H1299‐Control, and H1299‐SELENBP1) were harvested and fixed with fresh 70% alcohol at 4°C overnight. According to the manufacture instructions of Cell Cycle Kit (Yesen), all the cells were stained with propidium (PI) and incubated at 37°C for 30 min in the dark; next, the cell cycle was analyzed by FCM as soon as possible.

The indicated cells and their supernatant were harvested; most importantly, the cells were digested with EDTA‐free trypsin. According to the manufacture instructions of Annexin V‐Alexa Fluor 647/PI kit (Yesen), all cells were resuspended by 1 × Binging buffer; then, the 5 μL Annexin V‐Alexa Fluor 647 solution was added into 100‐μL cell suspension and mixed and incubated away from light at room temperature for 30 min; next, 10 μL PI staining solution and 400 μL PBS were added into above cell suspension, respectively. The cell apoptosis was analyzed by FCM in time.

### 2.12 Tumor xenograft model in nude mice

The animal experiments and all procedures involving the handling and treatment of mice during this study were approved by the Ethical Review Committees of West China Hospital, Sichuan University. All the experiments were performed according to the guidelines of the National Institutes of Health Guide for the Care and Use of Laboratory Animals. Twelve 4‐week‐old immunocompromised male nude mice were purchased from HFK Bioscience and used for xenograft studies. The stable SELENBP1‐overexpression A549 cells and control cells were digested to obtain single‐cell suspension. Each nude mouse was subcutaneously injected with 100 μL medium containing around 5 × 10^6^ cells. The tumor size was measured every 3 days after 5 days injection. All the mice were euthanized after 29 days. The volume of tumor (mm^3^) was calculated by: Volume = length × width^2^ × π/6. The half of all the transplanted tumors were placed in 4% paraformaldehyde and then embedded with paraffin, and the half of tumors were used to western blotting.

### 2.13 TUNEL assay

Paraffin tissue sections were deparaffinized and rehydrated. According to TdT‐mediated dUTP Nick‐End Labeling (TUNEL) assay kit (Yesen), add 100 μL fresh Proteinase K (2 μg/mL) solution and incubated at room temperature for 20 mins. Then, add 100 μL 1 × Equilibration Buffer (5 × Equilibration Buffer: ddH_2_O = 1:5) to each slice and incubated at room temperature for 20 min. Add 50 μL TdT incubation solution to each slide and incubated at 37°C for 60 min in the dark. Taken out the slide and incubated in PBS in room temperature and dark for 5 min, repeat twice. DAPI counterstain the nucleus, add DAPI dropwise, and incubated for 2 min in the dark. PBS wash two times, washed away excess DAPI, add mounting plate with antifluorescence quencher, mount the slide, take pictures under a Zeiss Imager Z2 microscope (Carl Zeiss; 200×).

### 2.14 Immunofluorescence (IF) assay

The cells (A549 and H1299) were seeded onto coverslips in 24‐well plates and cultured at 37°C incubator for 24 h, after cells were adhered on coverslips, taken out and fixed with 4% paraformaldehyde for 30 min, PBS wash 3 times, then permeabilized by fresh 0.5% Triton X‐100 and blocked with 1% bovine serum albumin (BSA). Immunofluorescence analysis assay was performed as described previously.^31^ Primary antibodies against SELENBP1 (1:200 dilution, Abcam, ab90135) and GPX1 (1:100 dilution, ABclonal, A1110), overnight at 4°C, followed by the detection with Alexa Fluor 488‐conjugated Goat Anti‐Rabbit IgG (H + L; 1:100 dilution, ABclonal, AS053) or Alexa Fluor 594‐conjugated Goat Anti‐Rabbit IgG (H + L; 1:100 dilution, ABclonal, AS039). Pictures were taken under a Zeiss Imager Z2 microscope (Carl Zeiss; 400×).

### 2.15 Statistical analysis

Data were analyzed with GraphPad Prism 7 or SPSS 22.0 software. Significant difference between two groups were analyzed using two‐tailed Student's *t*‐test. Multiple (three) groups were compared by using one‐way ANOVA. All data were presented as the Mean ± SD. The clinical statistic was analyzed by chi‐squared test. *p* < 0.05 (*), *p* < 0.01 (**), *p* < 0.005 (***), and *p* < 0.001 (****) were considered statistically significant.

## 3 RESULTS

### 3.1 SELENBP1 was downregulated in NSCLC tissues by a comprehensive analysis of TCGA database and collected clinical tissues

**FIGURE 1:**
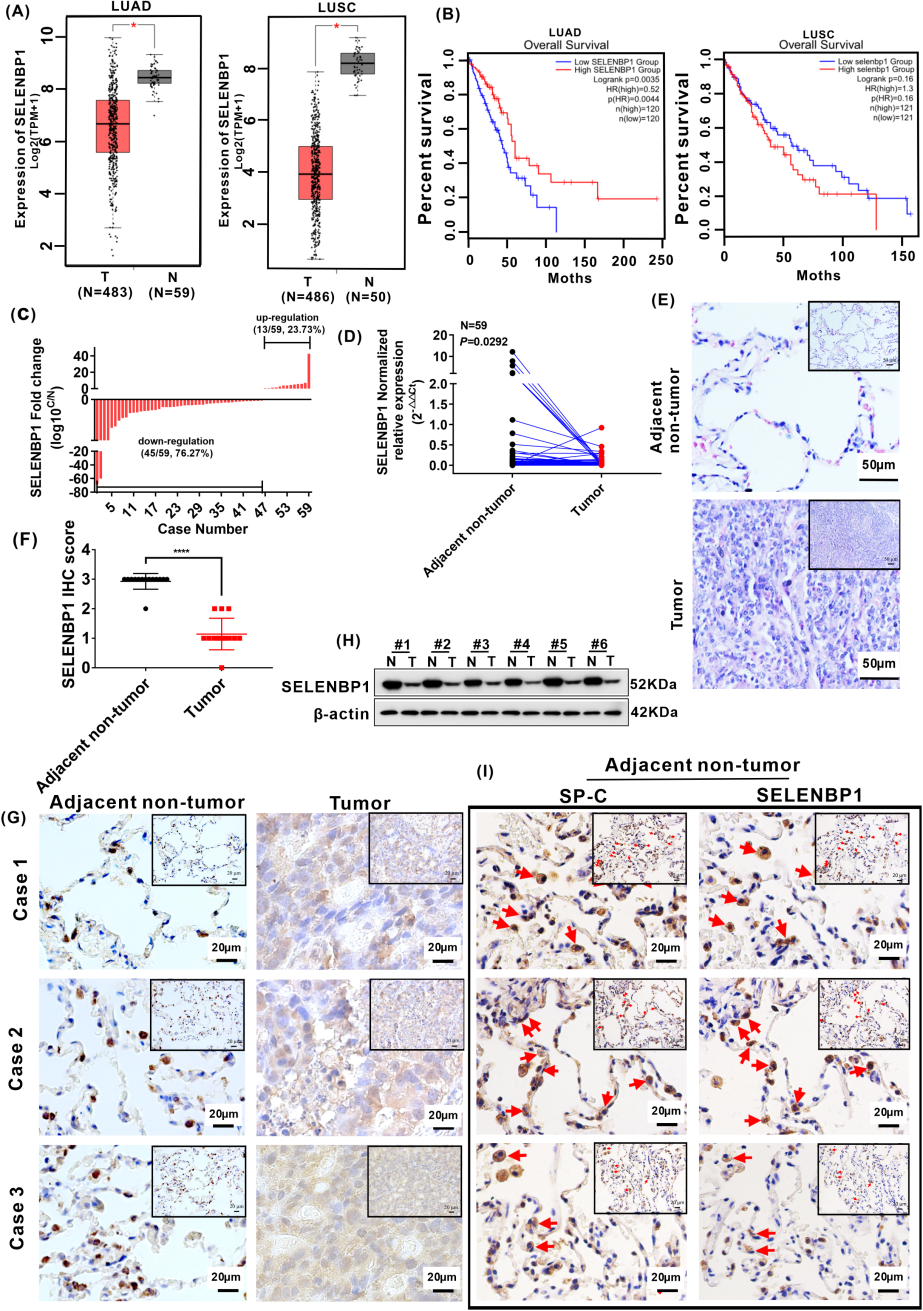
SELENBP1 was downregulated in NSCLC tissues by a comprehensive analysis of TCGA database and collected clinical tissues. (A) The expression levels of SELENBP1 in 483 LUAD tissues (T) and 59 adjacent nontumor tissues (N), 486 LUSC tissues (T), and 50 adjacent nontumor tissues (N; Data were obtained from TCGA database) “*” *p* < 0.05. T versus N in both LUAD and LUSC, respectively. (B)The relationship between the overall survival (OS) rate of LUAD tissues (*N* = 240), LUSC tissues (*N* = 242), and the expression levels of SELENBP1, respectively. (C) The expression of SELENBP1 in 59 paired collected clinical tissues was arranged by log10 C/N. (C) the expression of SELENBP1 in NSCLC tumor tissues. N, the expression of SELENBP1 in paired adjacent nontumor tissues. (D) Total RNA was isolated from collected clinical NSCLC tumor tissues and paired nontumor tissues, and the expression of SELENBP1 was assessed by qRT‐PCR analysis (*N* = 59). Paired *t*‐test. (E) The histologic structure of NSCLC tumor tissues and paired adjacent nontumor tissues were evaluated by H&E staining (*N* = 14). Results of H&E staining were observed under a bright field microscope (×200), Scale bars, 50 μm. (F) The histogram was used to quantify the experimental results of IHC staining. Data were presented as the Mean ± SD, unpaired *t*‐test. “****” *p* < 0.001. Adjacent nontumor tissue versus tumor tissues. (G) The expressions of SELENBP1 in collected clinical NSCLC tumor tissues and paired adjacent nontumor tissues were evaluated by IHC staining (*N* = 14). Results of IHC staining were observed under a bright field microscope (×400), Scale bars, 20 μm. (H) The protein expression of SELENBP1 in collected clinical NSCLC tumor tissues and paired adjacent nontumor tissues were detected by western blotting (*N* = 6). (I) The serial paraffin sections of the paired adjacent nontumor tissues were subjected to IHC staining with antibodies against SP‐C and SELENBP1 (*N* = 3). Staining without primary antibody was used as negative controls. All results were observed under a bright field microscope (×400). Scale bars, 20 μm. Images are shown while representative positive stains are indicated by red arrows.

Firstly, we analyzed the expression of SELENBP1 in TCGA database and found that the expression of SELENBP1 was significantly downregulated in LUAD tissues and LUSC tissues compared with adjacent nontumor tissues (Figure 1A). Next, higher expression of SELENBP1 has significant positive correlation with longer overall survival time in LUAD (*p* = 0.0044), but no significance in LUSC (*p* = 0.16; Figure 1B). Moreover, the expression of SELENBP1 has no significant correlation between stage 1 and stage 4, stage 2 and stage 3, stage 2 and stage 4, stage 3, and stage 4 in both LUAD and LUSC, respectively (Figure S1).

To further explore the expression of SELENBP1 in collected clinical NSCLC tissues, 59 clinical NSCLC tissues and their matched adjacent nontumor tissues were detected by qRT‐PCR. The expression of SELENBP1 was notably decreased in 45 out of 59 (45/59, 76.27%) human primary NSCLC tissues compared with their paired adjacent nontumor tissues (Figure 1C,D; Table S3). Then, we analyzed the relationship between the expression of SELENBP1 and age, gender, lymph node metastasis, and T stage, respectively. But the expression levels of SELENBP1 had no significant relationship with age, gender, lymph node metastasis, and T stage in both LUAD and LUSC (Table 1). Next, the histologic structure and the expression difference in SELENBP1 were analyzed by using collected clinical tissue specimens from LUAD patients (*N* = 14) with stage T1–T4 disease who underwent surgery resection. H&E staining showed that the adjacent nontumor tissues had obvious alveoli histologic structure, but in tumor tissues, the nuclear size of tumor cells increased and chromatin appeared less compact compared with their adjacent nontumor tissue cells (Figure 1E). Notably, the expression of SELENBP1 was also decreased in tumor tissues compared with adjacent nontumor tissues by IHC staining (Figure 1F,G) and western blotting (Figure 1H). The IHC score criteria and outcomes of SELENBP1 are shown in (Table S4). Altogether, these data specified that the expression of SELENBP1 was markedly decreased in NSCLC tissues compared with adjacent nontumor tissues in TCGA database and collected clinical tissues.

**TABLE 1:** Clinicopathological features in 59 clinical NSCLC tissues.

Clinicopathological parameter	Total No.	SELENBP1 relative expression		*p‐*value
		Upregulated	Downregulated	
		*N* = 13	*N* = 45	
Age (years)				0.2472
≤60	32	9	23	
>60	26	4	22	
Gender				0.4579
F	26	7	19	
M	32	6	26	
Lymph node metastasis				0.3746
Y	33	6	27	
N	25	7	18	
T stage				0.6915
T1	24	6	18	
T2 + T3 + T4	34	7	27	

Abbreviations: F, female; M, male; N, no; Y, Yes.

### 3.2 SELENBP1 was obviously expressed in alveolar type 2 (AT‐II) cells

The airway and alveoli of the mammalian lung have diverse stem cells and progenitor cells; these cells are responsible for substituting anile or/and dead cells in each compartment during homeostasis and repair of lung.^33^, ^34^ In alveolus, AT‐II cells serve as the predominant epithelial progenitor to complete the self‐renewal of alveoli after injury.^35^ Lineage tracing in mice showed that AT‐II cells defined by their specific expression of surfactant protein C (SFTPC/SP‐C) are capable of long‐term self‐renewal and multipotent differentiation to alveolar type I (AT‐I) cells.^35^, ^36^ We shiningly discovered that SELENBP1 was obviously expressed in corner of alveoli during detecting the expression of SELENBP1 in collected clinical NSCLC tissues and their paired adjacent nontumor tissues by IHC (Figure 1F,G). In order to preliminarily explore the expression site of SELENBP1 in the alveoli, the IHC staining of SELENBP1 and SP‐C on serial sections of adjacent nontumor tissues was performed. Our results illustrated that SELENBP1 and SP‐C were colocalized in the AT‐II cells (Figure 1I). Thus, these results preliminarily indicated that SELENBP1 was obviously expressed in AT‐II cells, and it may play a significant role in the homeostasis maintenance of AT‐II cells.

### 3.3 Overexpression of SELENBP1 inhibited the proliferation, migration, and invasion of NSCLC cells in vitro

**FIGURE 2:**
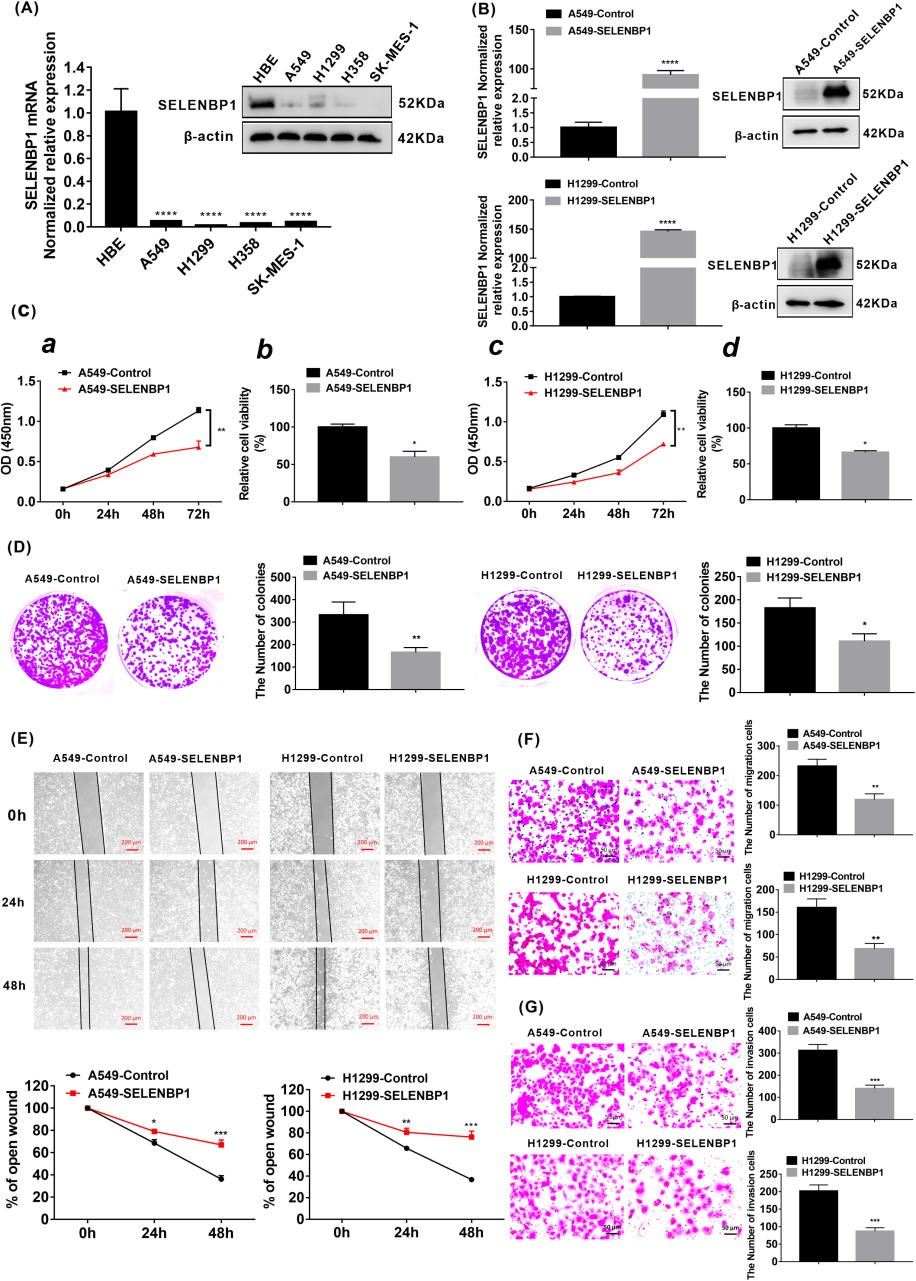
Overexpression of SELENBP1 inhibited the proliferation, migration, and invasion of NSCLC cells in vitro. (A) The expression of SELENBP1 in four NSCLC cell lines A549, H1299, H358, and SK‐MES‐1 and normal lung cells HBE were measured by qRT‐PCR analysis and western blotting. β‐Actin was used as a control. All data were presented as the mean ± SD, unpaired *t*‐test, “****” *p* < 0.001, HBE group versus A549 group, H1299 group, H358 group, and SK‐MES‐1 group, respectively. (B) The expression of SELENBP1 in both A549‐SELENBP1 cells and H1299‐SELENBP1 cells and their control cells were measured by qRT‐PCR and western blotting after Lentivirus infection treatment. The total RNA and protein were extracted from the cells after puromycin screening. All data were presented as the mean ± SD, unpaired *t*‐test, “****” *p* < 0.001, A549‐Control group versus A549‐SELENBP1 group, H1299‐Control group versus H1299‐SELENBP1 group. (C) CCK8 assay. (*a*, *c*) CCK8 was performed at 0, 24, 48 and 72 h (*N* = 5). All data were presented as the mean ± SD, unpaired *t*‐test, “**” *p* < 0.01, A549‐Control group versus A549‐SELENBP1 group, H1299‐Control group versus H1299‐SELENBP1 group. (*b*, *d*) The histogram was used to quantify the cell viability (%) in 72 h. All data were presented as the mean ± SD, unpaired *t*‐test, “*” *p* < 0.05. A549‐Control group versus A549‐SELENBP1 group and H1299‐Control group versus H1299‐SELENBP1 group. (D) The number of cell clone in the A549‐SELENBP1 and H1299‐SELENBP1 and the control group were measured by clone forming assay (*N* = 3). All data were presented as the mean ± SD, unpaired *t*‐test, “*” *p* < 0.05, “**” *p* < 0.01, A549‐Control group versus A549‐SELENBP1 group and H1299‐Control group versus H1299‐SELENBP1 group. (E) The rate of open wounds (%) was measured by wound healing analysis (*N* = 3), and all data were presented as the mean ± SD, unpaired *t*‐test, “**” *p* < 0.01, “***” *p* < 0.005, A549‐Control group versus A549‐SELENBP1 group and H1299‐Control group versus H1299‐SELENBP1 group. Scale bars, 200 μm. (F) The number of migrated cells in A549‐SELENBP1, H1299‐SELENBP1, and control group was measured by Millicell assay (*N* = 3). And data were presented as the mean ± SD, unpaired *t*‐test “**” *p* < 0.01, A549‐Control group versus A549‐SELENBP1 group and H1299‐Control group versus H1299‐SELENBP1 group. Scale bars, 50 μm. (G) The number of invasion cells was measured by Transwell assay (*N* = 3). The data were presented as the mean ± SD, unpaired *t*‐test “***” *p* < 0.005, A549‐Control group versus A549‐SELENBP1 group and H1299‐Control group versus H1299‐SELENBP1 group. Scale bars, 20 μm. All experiments were repeated at least three times.

Since the role of SELENBP1 in human NSCLC cells is still not clear; in this part, we first measured the expression profile of SELENBP1 in four NSCLC cell lines (A549, H1299, H358, and SK‐MES‐1) and human normal lung cells (HBE). The expression of SELENBP1 was significantly reduced in A549, H1299, H358, and SK‐MES‐1 compared with HBE by qRT‐PCR and western blotting (Figure 2A). Of note, A549 cells were originated from the malignant transformation of AT‐II cells.^37^, ^38^ Thus, A549 and H1299 cells were randomly chosen for follow‐up experiments. To further uncover the actions of SELENBP1 in human NSCLC cells, the stable SELENBP1‐overexpressing A549 and H1299 cell lines were established (named A549‐SELENBP1 and H1299‐SELENBP1), and the expression levels of SELENBP1 were quantified by qRT‐PCR and western blotting (Figure 2B). Then CCK8 assay (Figure 2C) and colony formation assay (Figure 2D) revealed that overexpression of SELENBP1 significantly suppressed the growth rate and tumorigenicity of both stable SELENBP1‐overexpressing cell lines compared with the control cells in vitro. In addition, the cell migration ability was evaluated by wound healing assay and Millicell assay, we found that the cell migration ability was significantly inhibited in A549‐SELENBP1 and H1299‐SELENBP1 cells compared with the control cells (Figure 2E,F). Furthermore, compared with the control cells, overexpression of SELENBP1 effectively inhibited the cell invasion ability by transwell assay (Figure 2G). Collectively, these results indicated that overexpression of SELENBP1 markedly inhibited the proliferation, migration, and invasion of NSCLC cells in vitro.

### 3.4 Overexpression of SELENBP1 suppressed the malignant progression of NSCLC cells via in part inactivating the PI3K/AKT/mTOR signaling pathway in vitro

**FIGURE 3:**
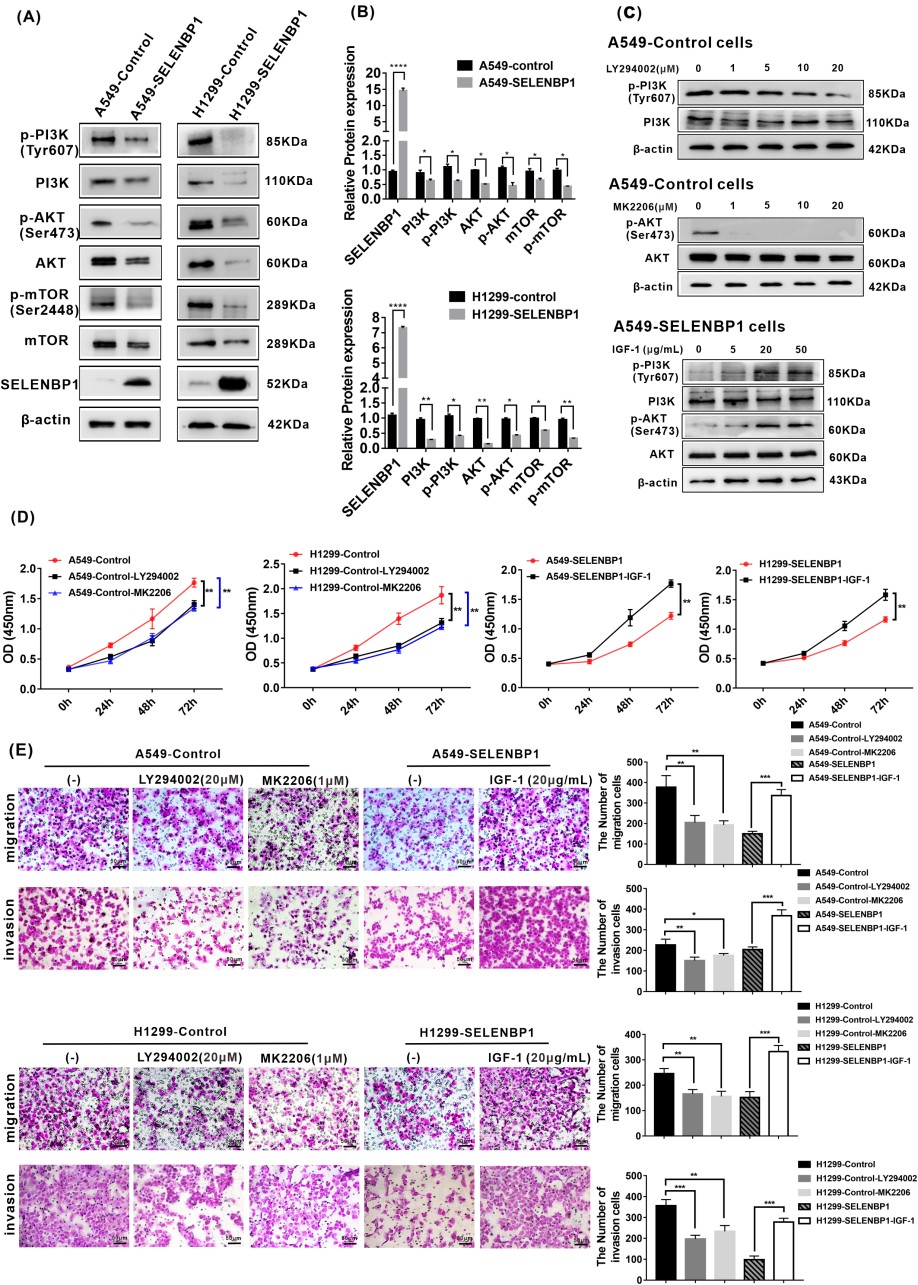
Overexpression of SELENBP1 suppressed the proliferation, migration, and invasion of NSCLC cells in part via inactivating the PI3K/AKT signaling pathway in vitro. (A) Western blotting analysis of PI3K/AKT/mTOR pathway markers in A549‐SELENBP1, H1299‐SELENBP1, and control cells. The NSCLC cell lysate was collected and subjected to western blotting analysis with antibodies against members of the PI3K/AKT/mTOR pathway. Levels of β‐Actin were used as loading control. (B) The histogram was used to quantify the experimental results of western blotting in A549‐SELENBP1 and their control cells, and H1299‐SELENBP1 and their control cells. All data were presented as the mean ± SD, unpaired *t*‐test, “*” *p* < 0.05, “**” *p* < 0.01, “****” *p* < 0.001, A549‐Control group versus A549‐SELENBP1 group and H1299‐Control group versus H1299‐SELENBP1 group. (C) Western blotting analysis of PI3K/mTOR pathway markers in the indicated cells treated with LY294002, MK‐2206, or IGF‐1 at various concentrations. (D) CCK8 was performed at 0, 24, 48 and 72 h in the indicated cells treated with LY294002 (20 μM), MK‐2206 (1 μM), or IGF‐1 (20 μg/mL; *N* = 4). And all data were presented as the mean ± SD. Multiple groups were compared by using one‐way ANOVA. Two groups were compared using unpaired *t*‐test “**” *p* < 0.01. A549‐Control group versus A549‐Control‐LY294002 group, A549‐Control group versus A549‐Control‐MK2206 group, A549‐SELENBP1 group versus A549‐SELENBP1‐IGF‐1 group. H1299‐Control group versus H1299‐Control‐LY294002 group, H1299‐Control group versus H1299‐Control‐MK2206 group, and H1299‐SELENBP1 group versus H1299‐SELENBP1‐IGF‐1 group. (E) The number of migrated cells and the number of invasion cells in indicated cells were measured by Millicell assay and transwell assay, respectively (left). Scale bars, 20 μm. The histogram was used to quantify the experimental results (right), and all data were presented as the mean ± SD. Multiple groups were compared by using one‐way ANOVA. Two groups were compared using unpaired *t*‐test “**” *p* < 0.01. “***” *p* < 0.005. A549‐Control group versus A549‐Control‐LY294002 group, A549‐Control group versus A549‐Control‐MK2206 group, A549‐SELENBP1 group versus A549‐SELENBP1‐IGF‐1 group. H1299‐Control group versus H1299‐Control‐LY294002 group, H1299‐Control group versus H1299‐Control‐MK2206 group, and H1299‐SELENBP1 group versus H1299‐SELENBP1‐IGF‐1 group.

Both selenium‐rich foods (containing organic and inorganic Se) and Se nanoparticles significantly inhibited the activation of PI3K/AKT/mTOR signaling pathway in cancers.^39^, ^40^ Importantly, the significant action of selenium is based on selenium‐containing protein as mediator.^10^ And previous studies have indicated that SELENBP1 performed an important role in the inhibition of cell proliferation in breast cancer and gastric cancer cells treated with selenium,^41^, ^42^ suggesting that SELENBP1 was one of the mediators for the antitumor effect of selenium. Furthermore, numerous oncogenes and growth factor receptors promote the activity of phosphoinositide 3 kinase (PI3K), and excessively elevated PI3K is regarded as a hallmark of cancer.^43^ Many studies verified that the activation of PI3K/AKT/mTOR signaling pathway played key actions in both tumorigenesis and progression of NSCLC.^44^, ^45^ Hence, based on the above studies, we hypothesized that the antitumor effect of SELENBP1 might be associated with the PI3K/AKT/mTOR signaling pathway in NSCLC. Then, we detected whether overexpression of SELENBP1 inhibited the malignant progression of NSCLC cells through the PI3K/AKT/mTOR signaling pathway. Notably, the most of essential members of PI3K/AKT/mTOR signaling pathway, such as PI3K, p‐PI3K, AKT, p‐AKT, mTOR, and p‐mTOR, were, respectively, downregulated in both A549‐SELENBP1 and H1299‐SELENBP1 compared with control cells by western blotting (Figure 3A,B). To further determine whether activation of PI3K/Akt/mTOR signaling was mediated by overexpression of SELENBP1 in NSCLC cells, we then investigated the effects of specific PI3K or Akt inhibitor and PI3K/AKT activator on the malignant characteristics of NSCLC cells, respectively. The effectiveness of different doses of PI3K inhibitor (LY294002), Akt inhibitor (MK‐2206), and PI3K/AKT activator (IGF‐1) was confirmed first (Figure 3C). The cell proliferation (Figure 3D), migration, and invasion (Figure 3E) of A549‐Control cells and H1299‐control cells were inhibited after treatment with the specific PI3K or Akt inhibitor. Meanwhile, the cell proliferation (Figure 3D), migration, and invasion (Figure 3E) of A549‐SELENBP1 cells and H1299‐SELENBP1 cells were promoted after treatment with a specific PI3K/AKT activator. Collectively, these data illustrated that overexpression of SELENBP1 significantly restrained the proliferation, migration, and invasion of NSCLC cells at least in part through inactivating the PI3K/AKT/mTOR signaling pathway in vitro.

Besides, we further analyzed the effect of SELENBP1 on cell cycle. The results discovered that overexpression of SELENBP1 induced an observably inhibition of S phase in both A549‐SELENBP1 cells and H1299‐SELENBP1 cells compared with their control cells (Figure S2A,B). It suggested that inhibition of S phase might be correlated with the tumor‐suppressive role of SELENBP1 in NSCLC cells in vitro.

### 3.5 Overexpression of SELENBP1 inhibiting the growth of NSCLC cells in vivo was associated with the inhibition of PI3K/AKT/mTOR pathway

**FIGURE 4:**
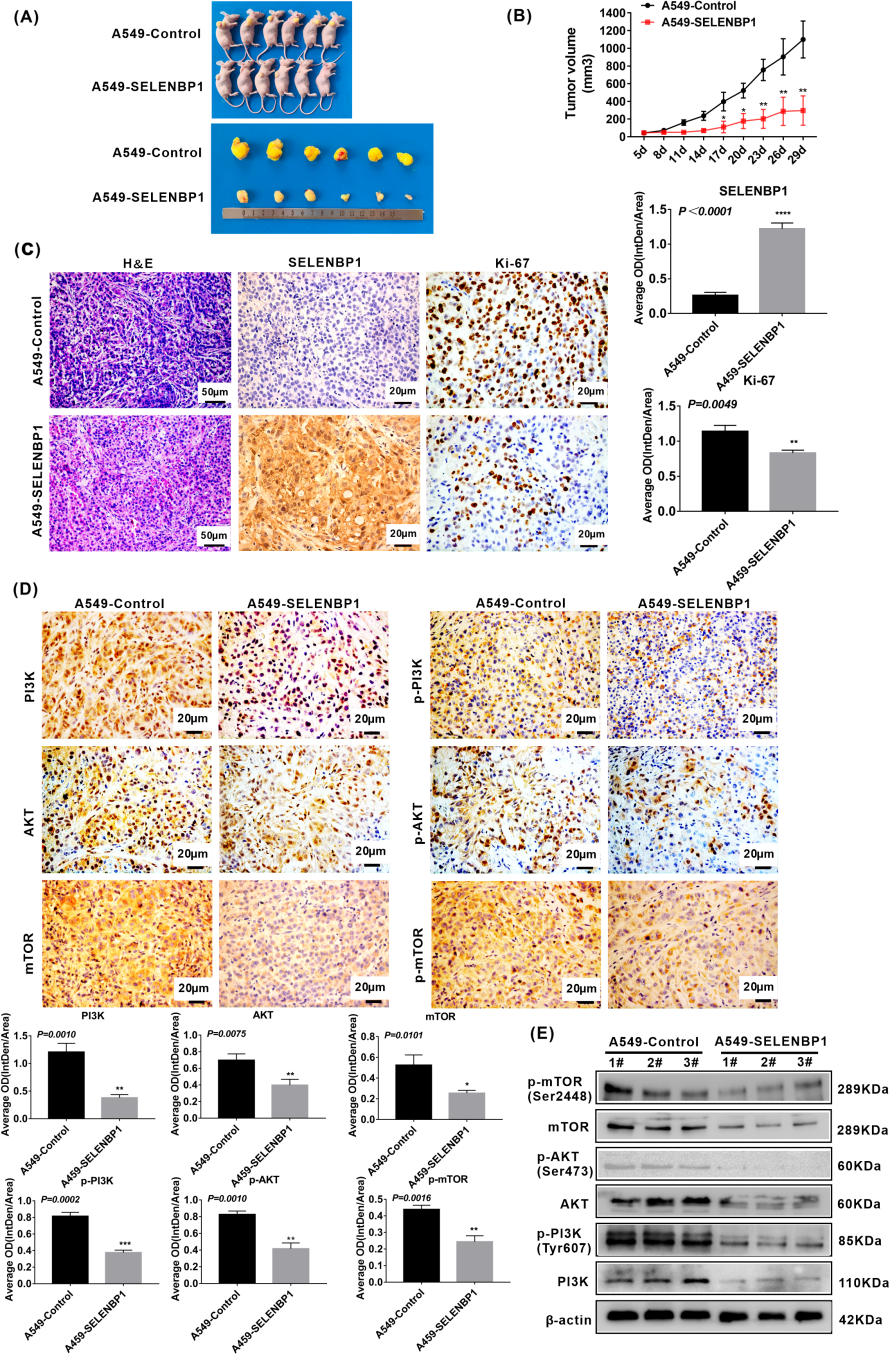
Overexpression of SELENBP1 inhibiting the growth of NSCLC cells in vivo was associated with the inhibition of PI3K/AKT/mTOR pathway. (A) Establishment of stable SELENBP1 overexpressing A549 cells and control cells tumor xenograft Model. Athymic nude mice (4‐week‐old male, *N* = 6/point/group) were subcutaneously injected A549‐SELENBP1 cells or A549‐Control cells, respectively. The mice were sacrificed after 29 days, the retrieved tumor samples were presented. (B) Volume of Xenograft tumor was measured once every 3 days after 5 days subcutaneous injection. Data were presented as the mean ± SD, unpaired *t*‐test, “*” *p* < 0.05, “**” *p* < 0.01, A549‐Control tumors group versus A549‐SELENBP1 tumors group. (C) Paraffin sections of the retrieved tumor samples were subjected to H&E staining and IHC staining with antibodies against SELENBP1, Ki‐67. Staining without primary antibody was used as negative controls. Results of H&E were observed under a bright field microscope (×200), Scale bars, 50 μm. Results of IHC were observed under a bright field microscope (×400), Scale bars, 20 μm. The histogram was used to quantify the experimental results of IHC (*N* = 3). All data were presented as the mean ± SD, unpaired *t*‐test, “**” *p* < 0.01, “****” *p* < 0.001, A549‐Control tumors group versus A549‐SELENBP1 tumors group. (D) Paraffin sections of the retrieved tumor samples were subjected to IHC staining with antibodies against PI3K, p‐PI3K, AKT, p‐AKT, mTOR, and p‐mTOR. Staining without primary antibody was used as negative controls. Results were observed under a bright field microscope (×400). Scale bars, 20 μm. The histogram was used to quantify the experimental results of IHC (*N* = 3). And all data were presented as the mean ± SD, unpaired *t*‐test, “*” *p* < 0.05, “**” *p* < 0.01, “***” *p* < 0.005, A549‐Control tumors group versus A549‐SELENBP1 tumors group. (E) The tumor tissues were retrieved, extracted total protein, and subjected to western blotting analysis of key components of PI3K/AKT/mTOR pathway (*N* = 3/group). Levels of β‐Actin were used as loading control.

To further confirm the function of SELENBP1 on the growth of NSCLC cells in vivo, we established a stable SELENBP1‐overexpressing A549 cells tumor xenograft model in athymic nude mice via subcutaneous injection as we previously reported.^31^ Experimentally, the tumor volume was measured once every 3 days after 5 days subcutaneous injection. The xenograft tumor volume was obviously inhibited in A549‐SELENBP1 tumors compared with A549‐Control tumors (Figure 4A,B). Then, tumor tissue samples were collected for further analyses. H&E staining illustrated that retrieved subcutaneous tumor tissues did not have any obviously histologic changes in both A549‐SELENBP1 tumors and A549‐Control tumors. Of note, the cell proliferation protein Ki‐67 was decreased in A549‐SELENBP1 tumors compared with A549‐Control tumors (Figure 4C). Furthermore, overexpression of SELENBP1 induced the inactivation of PI3K/AKT/mTOR signaling, the expression of PI3K, p‐PI3K, AKT, p‐AKT, mTOR, and p‐mTOR were decreased in A549‐SELENBP1 tumors compared with A549‐Control tumors by IHC staining (Figure 4D). We further analyzed the protein expression levels of the pivotal factors of PI3K/AKT/mTOR signaling axis by western blotting. Consistent with our results in vitro, the expression of PI3K, p‐PI3K, AKT, p‐AKT, mTOR, and p‐mTOR were also repressed in A549‐SELENBP1 tumors compared with A549‐Control tumors (Figure 4E). In total, these data indicated that overexpression of SELENBP1 significantly inhibited the growth of NSCLC cells in vivo, and it was associated with the inhibition of PI3K/AKT/mTOR pathway.

### 3.6 Overexpression of SELENBP1 inducing the apoptosis of NSCLC cells under nonhigh level of oxidative stress was associated with the activation of caspase‐3 signaling pathway in vitro

**FIGURE 5:**
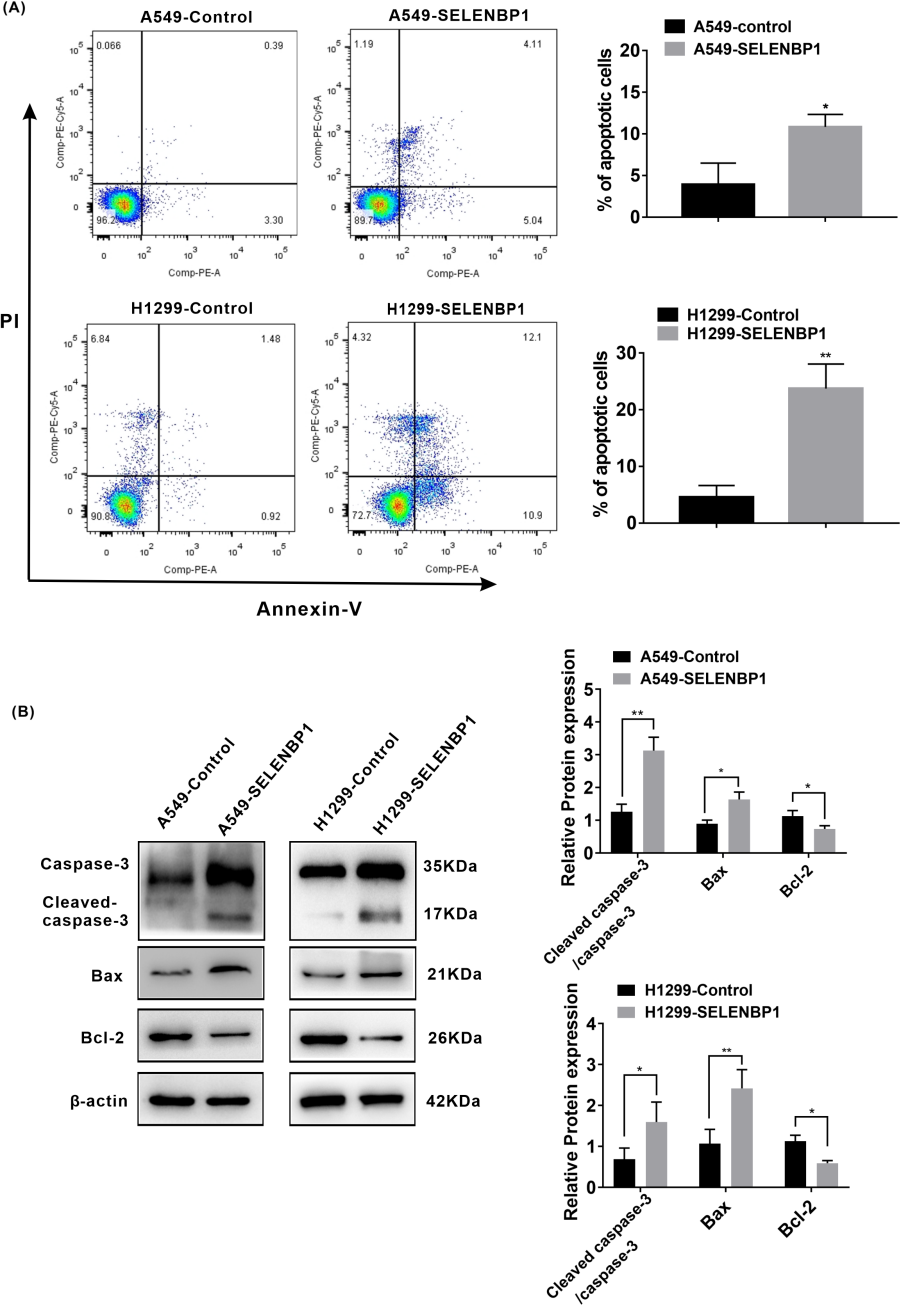
Overexpression of SELENBP1 inducing the apoptosis of NSCLC cells under nonhigh level of oxidative stress was associated with the activation of caspase‐3 signaling pathway in vitro. (A) Flow cytometry analysis was conducted (left), and the ratio of apoptotic cells (%) was calculated (*N* = 3; right). All data were presented as the mean ± SD, unpaired *t*‐test, “*” *p* < 0.05, “**” *p* < 0.01, A549‐Control group versus A549‐SELENBP1 group and H1299‐Control group versus H1299‐SELENBP1 group. (B) The effect of SELENBP1 overexpressing on the expression of the components of caspase‐3 pathway was measured. A549‐SELENBP1, H1299‐SELENBP1, and their control cells lysate were collected and subjected to western blotting analysis with antibodies against the caspase‐3, cleaved‐caspase‐3, Bcl‐2, and Bax (left). Levels of β‐Actin were used as loading control. The histogram was used to quantify the experimental results of western blotting (*N* = 3; right). All data were presented as the mean ± SD, unpaired *t*‐test, “*” *p* < 0.05, “**” *p* < 0.01, A549‐Control group versus A549‐SELENBP1 group and H1299‐Control group versus H1299‐SELENBP1 group.

Previous studies pointed out that SELENBP1 could mediate cell apoptosis in liver cancer cells^25^ and gastric cancer cells,^42^ but the potential impact of SELENBP1 on cell apoptosis in NSCLC cells remains to be fully understood. To detect whether overexpression of SELENBP1 mediates cell apoptosis in NSCLC cells, A549‐SELENBP1, H1299‐SELENBP1, and their respective control cells were analyzed by flow cytometry (FCM). As shown in Figure 5A, the rate of apoptotic cells was increased in A549‐SELENBP1 cells and H1299‐SELENBP1 cells compared with these of the control cells under nonhigh level of oxidative stress.

Bcl‐2 family proteins either have promoting or inhibiting apoptotic activities, in which, Bcl‐2 serves as a master antiapoptosis factor, but Bax is a pro‐apoptosis in cell apoptosis progression.^46^ Bcl‐2 family proteins regulate cell apoptosis by modulating mitochondrial membrane permeability. Following apoptotic signal, Bcl‐2 family proteins translocate to mitochondria, in which, pro‐apoptosis protein such as Bax lead to release of cytochrome C. Cytochrome C can activate caspase‐9, then activate caspase‐3, and finally generate cell apoptosis.^47^ The caspase‐3 is located at the end of the caspase pathway, and it is stimulated by both intrinsic and extrinsic death pathways in apoptosis progression compared with other members of the caspase family.^48^, ^49^ The activated caspase‐3 results in plasma membrane leakage, DNA cleavage, chromatin condensation, and ectropion of phosphatidylserine.^50^ To discover the mechanism of SELENBP1 on apoptosis in NSCLC cells, we measured the protein expression levels of the key proteins in apoptotic signaling pathway. As shown in Figure 5B, the essential members of apoptosis promoting factors such as caspase‐3, cleaved‐caspase‐3, and Bax were increased, whereas the apoptosis‐inhibiting factor Bcl‐2 was decreased in A549‐SELENBP1 and H1299‐SELENBP1 compared with their control cells by western blotting. Hence, these results indicated that overexpression of SELENBP1 induced the apoptosis of NSCLC cells under nonhigh level of oxidative stress in vitro, which was associated with the activation of caspase‐3 signaling pathway.

### 3.7 Overexpression of SELENBP1 inducing the apoptosis of NSCLC cells under high level of oxidative stress might be related to its combining with GPX1 and colocalizing in nucleus in vitro

Furthermore, the increased reactive oxygen species (ROS) level could activate antitumor signaling, which caused oxidative stress induced‐cancer cell apoptosis.^51^ To assess whether overexpression of SELENBP1 also mediate apoptosis of NSCLC cells under high level of oxidative stress, A549‐SELENBP1, H1299‐SELENBP1, and their control cells were treated with hydrogen peroxide treatment (200 μM) for 24 h, and then, FCM analysis performed that the rate of apoptotic cells was increased in both of A549‐SELENBP1 and H1299‐SELENBP1 group compared with their control groups (Figure S3A). Therefore, these data revealed that overexpression of SELENBP1 could also induce the apoptosis of NSCLC cells under high level of oxidative stress.

Moreover, to detoxify increased ROS levels, establish a redox balance, and resist to apoptosis, the expression of antioxidant proteins was increased in cancer cells.^51^ Considering the previous studies, the antioxidant enzyme GPX1 (glutathione peroxidase 1), which contains selenocysteine, is widely expressed and protects cells from ROS and hydrogen peroxide‐induced or dependent apoptosis.^52^, ^53^, ^54^ However, aberrantly increased expression of GPX1 in many cancers is closely connected to tumorigenesis and progression including LC.^55^ To further determine whether overexpression of SELENBP1 could promote apoptosis by regulating the expression of GPX1 under high level of oxidative stress, the mRNA and protein expression of GPX1 was detected in both A549‐SELENBP1 and H1299‐SELENBP1 compared with their control cells following hydrogen peroxide treatment (200 μM) for 24 h. We found that the mRNA and protein expression of GPX1 did not be affected by overexpression of SELENBP1 under high level of oxidative stress by qRT‐PCR and western blotting (Figure S4).

Besides, Huang et al.^25^ discovered that the combination of SELENBP1 and GPX1 in nucleus could inhibit activity of GPX1, which induced apoptosis of HCC cells under high levels of oxidative stress. Based on the above results, cellular immunofluorescence (IF) staining was conducted to detect whether overexpression of SELENBP1 could change the localization of GPX1 from cytoplasm to nucleus in NSCLC cells. Because both stable SELENBP1‐overexpressing cells and control cells were carried with green fluorescence, A549 and H1299 were chosen in order to eliminate the effect of the green fluorescence on the experimental results. Our results revealed that SELENBP1 and GPX1 might colocalize in nucleus following hydrogen peroxide treatment for 24 h in A549 and H1299 cells (Figure S3B). Altogether, these data suggested that overexpression of SELENBP1 induced the apoptosis of NSCLC cells under high level of oxidative stress, and it might be related to SELENBP1 combining with GPX1 and colocalizing in nucleus.

### 3.8 Overexpression of SELENBP1 promoting the apoptosis of NSCLC cells in vivo was associated with the activation of caspase‐3 signaling pathway

**FIGURE 6:**
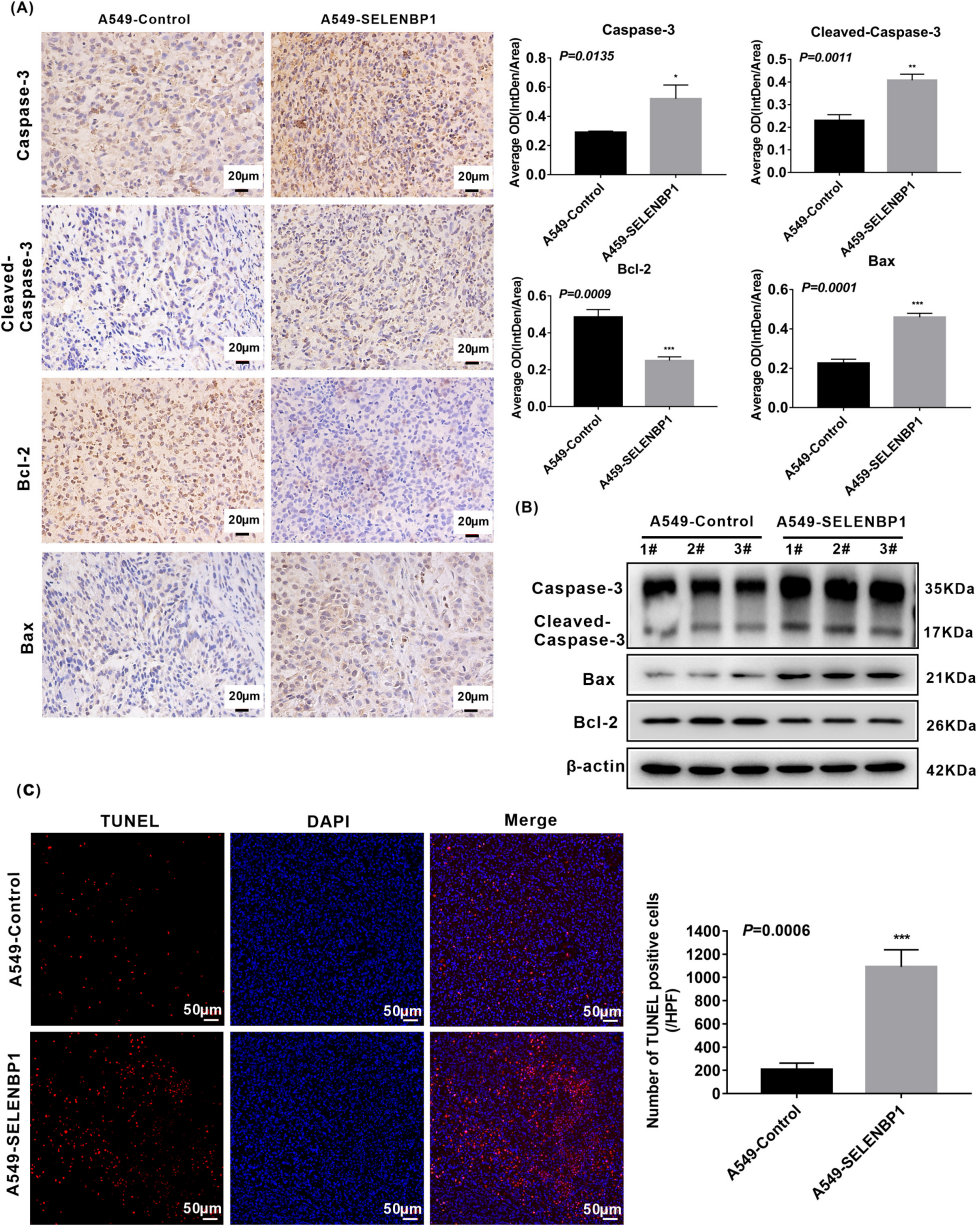
Overexpression of SELENBP1 promoting the apoptosis of NSCLC cells in vivo was associated with the activation of caspase‐3 signaling pathway. (A) Paraffin sections of the retrieved tumor samples were subjected to IHC staining with antibodies against caspase‐3, cleaved‐caspase‐3, Bcl‐2, and Bax (left). Staining without primary antibody was used as negative controls. Results were observed under a bright field microscope (×400). Scale bars, 20 μm. The histogram was used to quantify the experimental results of IHC (*N* = 3; right). All data were presented as the mean ± SD, unpaired *t*‐test, “*” *p* < 0.05, “**” *p* < 0.01, “***” *p* < 0.005, A549‐Control tumors group versus A549‐SELENBP1 tumors group. (B) The expression of caspase‐3, cleaved‐caspase‐3, Bcl‐2, and Bax in mice tumor xenograft model were measured by western blotting (*N* = 3/group). Levels of β‐Actin were used as loading control. (C) The lever of apoptosis was presented by TUNEL staining (*N* = 3; left), and the histogram was used to quantify the TUNEL‐positive cells (right). Images were observed under a microscope (×200). Scale bars, 50 μm. TUNEL‐stained cells are in red, and DAPI‐stained nuclei are in blue. And the data were presented as the mean ± SD, unpaired *t*‐test, “***” *p* < 0.005, A549‐Control tumors group versus A549‐SELENBP1 tumors group.

To further verify the function of SELENBP1 on apoptosis of NSCLC cells in vivo, the above A549‐SELENBP1 subcutaneous tumor model established previously was also used. In accordance with the above results in vitro, IHC staining of caspase‐3, cleaved‐caspase‐3, and Bax were also significantly increased, whereas Bcl‐2 was decreased in A549‐SELENBP1 tumors compared with A549‐Control tumors (Figure 6A). Accordingly, the elevated expression levels of caspase‐3, cleaved‐caspase‐3, Bax, and the decreased expression of Bcl‐2 were also observed in A549‐SELENBP1 tumors compared with A549‐Control tumors by western blotting (Figure 6B). Moreover, TUNEL assay demonstrated that TUNEL positive rate was augmented in A549‐SELENBP1 tumors compared with A549‐control tumors (Figure 6C). Collectively, these evidences suggested that overexpression of SELENBP1 facilitated the apoptosis of NSCLC cells in vivo, and it was associated with the activation of caspase‐3 signaling pathway.

## 4 DISCUSSION

Up to now, the previous studies have established an initial connection between SELENBP1 and human cancers, the expression of SELENBP1 was decreased in human cancer tissues including NSCLC, and the downregulated expression of SELENBP1 was associated with the progression and poor prognosis.^11^, ^17^ However, a few studies reported that the expression of SELENBP1 was decreased in most of the NSCLC tissues compared with adjacent nontumor tissues, and it was associated with poor outcomes.^28^, ^29^ Furthermore, only Deborah R et al.^30^ reported that SELENBP1 could inhibit growth and metastasis of tumors in Kras^G12D^‐driven mice lung adenocarcinoma model. Nevertheless, the biological functions and mechanisms of SELENBP1 in human NSCLC have not yet been ascertained in any detail until now. In this study, we identified the effects and the potential mechanisms of SELENBP1 in human NSCLC cells. It will help to deeply understand the underlying molecular pathogenesis of NSCLC and provide a theoretical basis for early clinical diagnosis and molecular therapy strategies.

Firstly, we analyzed the expression levels of SELENBP1 in NSCLC tissues and adjacent nontumor tissues in TCGA database. The Cancer Genome Atlas (TCGA) is a database that integrates information from around 11,000 individuals who bear over 30 different types of cancer, providing an information platform for large‐scale clinical data extraction and analysis.^56^ We found that the expression levels of SELENBP1 were decreased in both LUAD tissues and LUSC tissues compared with adjacent nontumor tissues in TCGA database. Consistent with our acquired data from TCGA database, Chen et al.^29^ pointed that the expression of SELENBP1 was downregulated in 92 human LUAD tissues compared with 10 adjacent nontumor tissues by 2D‐PAGE and 2D‐western blotting. And Tan et al.^28^ found that the expression of SELENBP1 was downregulated in 66 LUSC tissues compared with their paired adjacent nontumor tissues by IHC. In the current study, the expression of SELENBP1 was also significantly downregulated in 45/59 cases of collected NSCLC tissues relative to their paired adjacent nontumor tissues (in 38/49 of LUAD and in 7/10 of LUSC), which further demonstrated that the expression of SELENBP1 was decreased in the most of human primary NSCLC tissues relative to their paired adjacent nontumor tissues. Most importantly, in our study, the expression levels of SELENBP1 had no significant relationship with disease stage in both LUAD and LUSC from TCGA database and collected clinical NSCLC tissues, which hinted that SELENBP1 might be an early gene in the development of NSCLC. Meanwhile, we revealed that the patient with higher SELENBP1 levels experienced longer periods of overall survival (OS) in TCGA database. Due to the number of collected clinical tissues was limited, the expression levels of SELENBP1 had no significant relationship with lymph node metastasis in our collected clinical NSCLC tissues. More clinical tissues will be collected to further investigate the issue. The latest study has also established a convergence between a higher expression of SELENBP1 and a lower risk of patient death in LUAD.^5^ Both our results from TCGA database and other previous study reminded that the expression of SELENBP1 may also be a potential biomarker for predicting prognosis of NSCLC.

Furthermore, we also unexpectedly uncovered that SELENBP1 was visibly expressed in corner of alveolar during detecting the expression of SELENBP1 in collected clinical NSCLC tissues and their paired adjacent nontumor tissues by IHC, and these cells were with the positive expression of SP‐C. SP‐C is a specific marker of AT‐II cells.^36^ Our results preliminarily indicated that SELENBP1 was obviously expressed in AT‐II cells. Alveoli, the terminal respiratory units of lung, are tiny sac‐like structure where oxygen enters the bloodstream through squamous AT‐I cells and cuboidal AT‐II cells.^57^, ^58^ Of these, AT‐II cells are considered as bifunctional progenitor cells in alveoli for a long time, and it performs a crucial role in secreting phospholipid surfactants such as SP‐C for preventing the collapse of alveoli during respiration. Except to self‐renewal, AT‐II cells can also differentiate into AT‐I cells, which providing a large surface area to promote gas exchange.^57^, ^58^, ^59^ The previous studies have pointed to AT‐II cells as the predominant cells in tumorigenesis of LUAD and LUSC.^57^, ^60^, ^61^ In the current study, we uncovered that SELENBP1 was obviously expressed in AT‐II cells for the first time, but SELENBP1 was downregulated in tumor tissues compared with their paired adjacent nontumor tissues. These data reminded that the downregulated expression of SELENBP1 might be associated with the origin and progresses of NSCLC. Therefore, a better understanding for the action and mechanisms of SELENBP1 may help to reveal the origin and development of NSCLC.

However, a little research reported on the biological effects and molecular mechanisms of SELENBP1 in human NSCLC cells. Up to now, to our knowledge, only one study demonstrated that the downregulated expression of SELENBP1 could notably increase the clonal growth and migration of mice lung adenocarcinoma cells 394 T4 in vitro, and promote 394 T4 cells growth in vivo.^30^ Therefore, we respectively detected the mRNA and protein expression of SELENBP1 in four human NSCLC cell lines (A549, H1299, H358, and SK‐MES‐1) and normal lung cells HBE. Compared with HBE, the expressions of SELENBP1 were significantly downregulated in A549, H1299, H358, and SK‐MES‐1 cell lines. Of note, A549 cells were originated from the malignant transformation of AT‐II cells;^37^, ^38^ then, A549 and H1299 cells were randomly chosen for follow‐up experiments. And the stable SELENBP1‐overexpressing A549 and H1299 cell lines and control cell lines were established by lentivirus infection. In our study, overexpression of SELENBP1 markedly inhibited the proliferation, migration, and invasion of NSCLC cells in vitro, and further experiments in vivo confirmed that overexpression of SELENBP1 could also inhibit the growth of tumor cells. Similarly, the previous studies reported that overexpression of SELENBP1 suppressed the malignant characteristics of colorectal cancer cells^24^ and prostate cancer cells^23^ in vitro and/or in vivo. And downregulated expression of SELENBP1 increased cell proliferation and migration in liver cancer cells SMMC7721.^25^

Multitudinous oncogenes and growth factor receptors promote the activity of phosphoinositide 3 kinase (PI3K), and excessively elevated PI3K is regarded as a hallmark of cancer.^43^ The abnormal overexpression or activation of AKT has been reported in many human cancers including NSCLC, which is directly connected to enhanced cancer cell survival and proliferation.^62^ The mechanistic target of rapamycin (mTOR) is a kinase, which is regulated by anabolic signals, and the mTOR plays vital effects in regulating the proliferation and metabolism of cells.^43^, ^63^ Furthermore, the growing evidences confirmed that the activation of the PI3K/Akt/mTOR pathway could promote the proliferation and metastasis of NSCLC cells.^44^, ^45^, ^64^ Therefore, we speculated that the overexpression of SELENBP1 inhibiting the malignant progression of NSCLC cells might be connected to the inactivation of PI3K/AKT/mTOR pathway. Indeed, we uncovered that overexpression of SELENBP1 decreased the expression of PI3K, p‐PI3K, AKT, p‐AKT, mTOR, and p‐mTOR in A549‐SELENBP1 and H1299‐SELENBP1 compared with their control cells, respectively. Our data further indicated that the cell proliferation, migration, and invasion of A549‐Control cells and H1299‐Control cells were inhibited after treatment with the specific PI3K or Akt inhibitor. Meanwhile, the cell proliferation, migration, and invasion of A549‐SELENBP1 cells and H1299‐SELENBP1 cells were promoted after treatment with a specific PI3K/AKT activator. In addition, we also verified that overexpression of SELENBP1 inhibited the growth of tumor cells in vivo, and it was correlated with the inhibition of PI3K/AKT/mTOR pathway. Consequently, our data demonstrated that overexpression of SELENBP1 inhibited the malignant process of NSCLC at least in part via inactivating the PI3K/AKT/mTOR pathway. However, the reasons for changes in total protein levels and how these changes affect biological functions need to be investigated in the future. Of course, other pathways will be further explored in the future. Moreover, in the current study, we also discovered that overexpression of SELENBP1 weakened cell proliferation by inhibiting the S‐phase progression of A549‐SELENBP1 and H1299‐SELENBP1 in vitro. It implied that overexpression of SELENBP1 might be involved in inhibiting cell cycle progression by inhibiting cellular DNA replication. Similarly, in the previous study, SELENBP1 was also reported as an inhibitor in the cell cycle of bladder cancer cells, and the potential mechanisms had been shown that ectopic expression of SELENBP1 was indispensable for SELENBP1‐mediated transcriptional induction of p21.^26^ Certainly, the mechanisms of SELENBP1 regulating cell cycle of NSCLC cells need to be further explored in the future.

In addition, the previous studies pointed out that SELENBP1 could mediate cell apoptosis in liver cancer cells^25^ and gastric cancer cells,^42^ but the potential impact of SELENBP1 on cell apoptosis in NSCLC cells remains to be fully understood. Apoptosis is a noninflammatory type of programmed cell death (PCD), it is regulated by activating the apoptotic caspases and takes place through either intrinsic or extrinsic apoptotic pathway.^65^ The caspase‐3 is located at the end of the caspase pathway, and it is stimulated by both intrinsic and extrinsic death pathways in apoptosis progression compared with other members of the caspase family.^48^, ^49^ The activated caspase‐3 results in plasma membrane leakage, DNA cleavage, chromatin condensation, and ectropion of phosphatidylserine.^50^ Bcl‐2 family proteins have either promoting or inhibiting apoptotic activities, in which, Bcl‐2 serves as a master antiapoptosis factor, but Bax is a pro‐apoptosis in cell apoptosis progression.^46^ In our report, overexpression of SELENBP1 was shown to induce apoptosis of NSCLC cells under nonhigh level of oxidative stress. Moreover, overexpression of SELENBP1 indeed significantly increased the levels of caspase‐3, cleaved‐caspase‐3, Bax, and decreased the levels of Bcl‐2 in vitro and in vivo. In brief, overexpression of SELENBP1‐inducing cell apoptosis under nonhigh level of oxidative stress was correlated with the activation of caspase‐3 signaling pathway.

Furthermore, the elevated ROS level could activate antitumor signaling, which led to oxidative stress‐induced‐cancer cell apoptosis.^51^ To neutralize the elevated ROS level, establish a redox balance and resist to apoptosis, the expression levels of antioxidant proteins were increased in cancer cells.^51^ As the first discovered selenium‐containing protein in 1973, selenocysteine‐containing antioxidant enzyme GPX1 detoxifies hydroperoxides by reducing equivalents from glutathione.^66^ Particularly, selenocysteine was considered as a key component in regulation of GPX1 enzymatic activity. Antioxidant GPX1 was reported that it played a vital role in normal cell growth and adaptive pathological reaction, such as apoptosis or inflammation.^67^ However, the aberrantly increased expression levels of GPX1 in many cancers were closely related to tumorigenesis and progression.^55^ Previous researches had already revealed that the abnormally increased GPX1 activity could decrease tumor sensitivity toward ROS‐generating anticancer drugs.^68^, ^69^ The activation of GPX1 allowed the intense oxidative stress in the tumor microenvironment to be reduced, which allowed cancer cells to survive, proliferation, malignant transformation, and metastasis of cancer cells.^70^ In this study, our data revealed that overexpression of SELENBP1 promoted cell apoptosis under high level of oxidative stress. Moreover, consistent with the previous report in HCC cells,^25^ we found that the mRNA and protein levers of GPX1 did not be affected by overexpression of SELENBP1 under high level of oxidative stress. Of note, Huang et al.^25^ discovered that the GPX1 activity could also be alleviated by the combination of SELENBP1 and GPX1 in nucleus in HCC cells, which induced apoptosis of HCC cells under high levels of oxidative stress. This result hinted that the overexpression of SELENBP1 inducing the apoptosis of NSCLC cells might also be associated with the localization of GPX1 from cytoplasm to nucleus. In this study, we discovered that SELENBP1 might effectively block the antioxidant effect of GPX1 in the microenvironment of high oxidative stress, and it might be related to SELENBP1 combining with GPX1 and colocalizing in the nucleus. Consequently, according to our results and the previous study, we hypothesized that SELENBP1 altered the localization of GPX1 from cytoplasm to nucleus in cells, which might be responsible for SELENBP1 inducing apoptosis under high level of oxidative stress. However, the association between SELENBP1‐GPX1 complex and cell apoptosis needs to be further explored in the future, for instance, whether SELENBP1 is a key element for altering the localization of GPX1 in NSCLC cells.

Besides, the enhancive ROS could directly influence caspase pathway both in the intrinsic and in the extrinsic apoptotic pathways, and excessive ROS could also impact the apoptotic effectors including the Bcl‐2 family proteins.^51^ In our study, further experiments in vivo also verified that overexpression of SELENBP1 induced the apoptosis of NSCLC cells, and it was correlated with the activation of caspase‐3 signaling pathway. However, due to the restriction of experimental methods, the localization of GPX1 from cytoplasm to nucleus under high levels of oxidative stress could not been confirmed in vivo. Certainly, the specific factor mediated by overexpression SELENBP1 in cell apoptosis progression remains to be elucidated in future studies.

## 5 CONCLUSION

**FIGURE 7:**
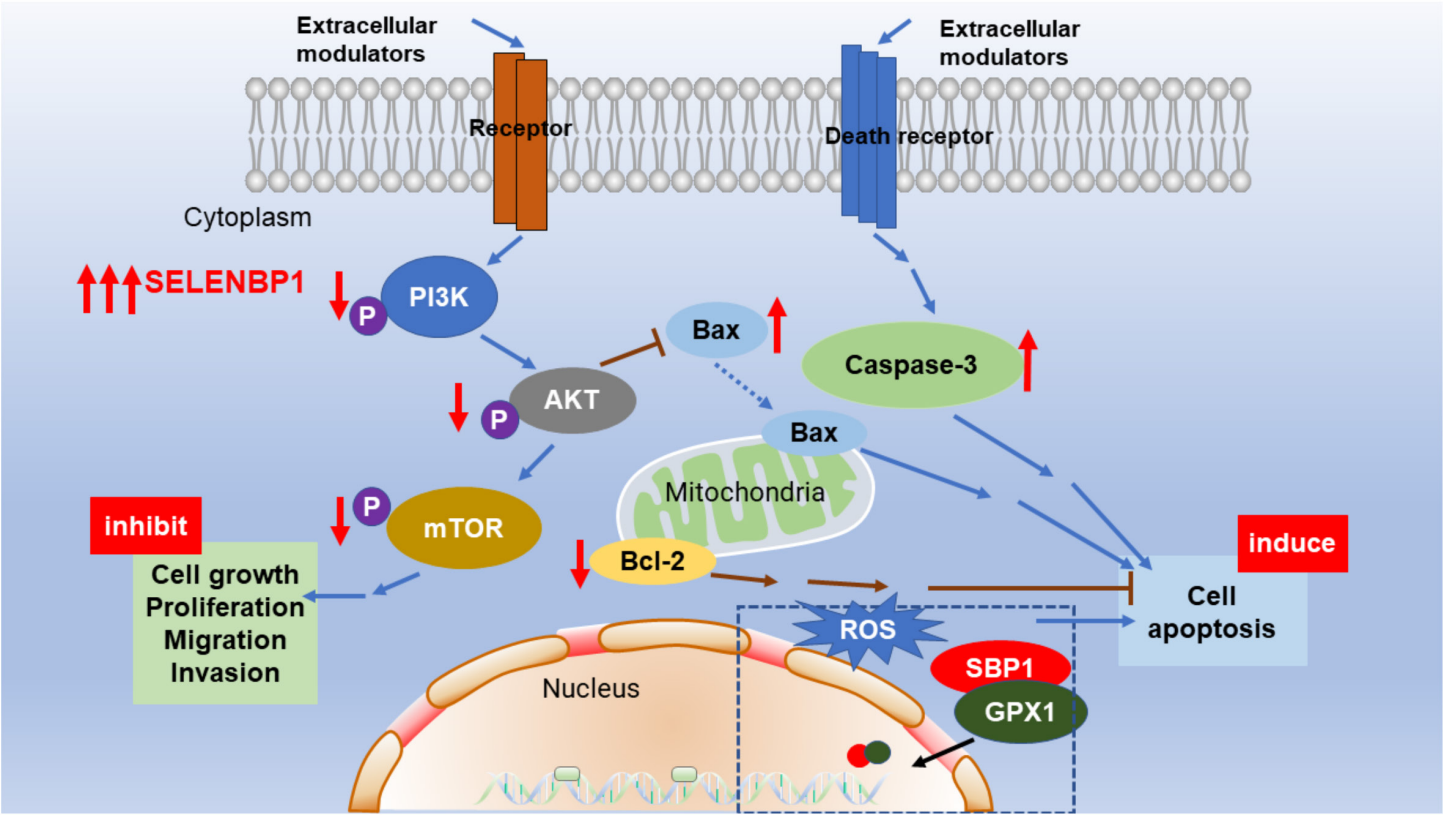
Molecular mechanisms for overexpression of SELENBP1 inhibiting the malignant progression and inducing the apoptosis in NSCLC cells. Overexpression of SELENBP1 decreased the expression of PI3K, p‐PI3K, AKT, p‐AKT, mTOR, p‐mTOR, and Bcl‐2, whereas increased the expression of Bax and caspase‐3. Overexpression of SELENBP1 suppressed the malignant progression of NSCLC cells by inhibiting the PI3K/AKT/mTOR signaling. Moreover, overexpression of SELENBP1 inducing the apoptosis of NSCLC cells was associated with the activation of caspase‐3‐dependent axis under nonhigh level of oxidative stress. Furthermore, overexpression of SELENBP1 facilitating the cell apoptosis under high level of oxidative stress was related to its combining with GPX1 and colocalizing in nucleus (ROS, reactive oxygen species).

In summary, we investigated the clinical impact, functional significance, and molecular mechanisms of SELENBP1 in human NSCLC. And we confirmed that overexpression of SELENBP1 inhibited the malignant progression and induced the cell apoptosis via distinct mechanisms in NSCLC (Figure 7). Particularly, we found that SELENBP1 and SP‐C were colocalized in the AT‐II cells for the first time, which preliminarily revealed that SELENBP1 was obviously expressed in the AT‐II cells. Therefore, our findings strongly indicated that SELENBP1 was an important tumor suppressor in the origin and development of NSCLC, and it may help to discover novel biomarkers or drug therapy targets for NSCLC. Besides, our study also make contribution for further understanding the antitumor mechanisms of selenium.

## AUTHOR CONTRIBUTIONS

**Ying Zhu:** Conceptualization (equal); data curation (lead); formal analysis (lead); investigation (lead); methodology (lead); software (lead); validation (lead); visualization (lead); writing – original draft (lead); writing – review and editing (equal). **Qiang Pu:** Data curation (supporting); investigation (supporting); resources (supporting); supervision (supporting). **Qiongyin Zhang:** Formal analysis (supporting); investigation (supporting); methodology (supporting); software (supporting); validation (supporting). **Yang Liu:** Formal analysis (supporting); investigation (supporting); methodology (supporting); software (supporting). **Yongfang Ma:** Investigation (supporting); methodology (supporting); software (supporting). **Yue Yuan:** Methodology (supporting); software (supporting); writing – original draft (supporting). **Lunxu Liu:** Project administration (supporting); resources (equal); supervision (supporting). **Wen Zhu:** Conceptualization (lead); funding acquisition (lead); methodology (supporting); project administration (lead); resources (lead); supervision (lead); writing – review and editing (lead).

## FUNDING INFORMATION

This reported study was supported by the Sichuan Provincial Department of Science and Technology/Applied Basic Research Program of Sichuan Province (No. 2018JY0134).

## CONFLICT OF INTEREST STATEMENT

None.

## ETHICS APPROVAL STATEMENT

Animal experiments of this study were approved by the Ethical Review Committees of West China Hospital, Sichuan University (Chengdu, China). All the experiments were performed according to the guidelines of the National Institutes of Health Guide for the Care and Use of Laboratory Animals. All clinical tissue samples and related anonymous clinical data were provided by the Institute of Thoracic Oncology and Department of Thoracic Surgery, West China Hospital, Sichuan University (Chengdu, China). All patients signed informed consent forms.

## Supporting information


Data S1

## Data Availability

The datasets used to support the finding of this study are available from the corresponding author and the first author upon reasonable request.
