# Supplementary material for: Selenium‐binding protein 1 inhibits malignant progression and induces apoptosis via distinct mechanisms in non‐small cell lung cancer
Source: Cancer Med. 2023 Aug 22;12(16):17149–70. doi: 10.1002/cam4.6309 (PMC10501285; doi:10.1002/cam4.6309)
Supplement: Supplementary file 1 — Data S1 [file CAM4-12-17149-s001.docx]

**APPENDIX**

**Figure S1.** The relationship between the expression of SELENBP1 and the disease stages of NSCLC in TCGA database.

**Figure S2.** Overexpression of SELENBP1 inhibited S phase of NSCLC cells.

**Figure S3.** Overexpression of SELENBP1 inducing the apoptosis of NSCLC cells under high level of oxidative stress might be related to its combining with GPX1 and colocalizing in nucleus in vitro.

**Figure S4.** The mRNA and protein expression of GPX1 did not be affected by overexpression of SELENBP1 under high level of oxidative stress.

**Table S1.** The clinicopathological features and relative expression of SELENBP1 in 59 clinical NSCLC tissues.

**Table S2.** The list of qRT-PCR primers.

**Table S3.** The relative expression of SELENBP1 in 59 clinical NSCLC tissues.

**Table S4.** The IHC score criteria and outcomes of SELENBP1

**Supplemental Table 1**

Table S1. The clinicopathological features and relative expression of SELENBP1 in 59 clinical NSCLC tissues

| NO. | Gender | Age | Pathology | Differentiation | N stage | T stage | The normalized expression of SELENBP1 in tumor tissues compared to paired adjacent non-tumor tissues |
| --- | --- | --- | --- | --- | --- | --- | --- |
| 1 | M | 50 | A | low | Y | T4 | 0.181 |
| 2 | M | 51 | A | low | Y | T1 | 0.838 |
| 3 | M | 54 | A | Medium-low | Y | T4 | 0.303 |
| 4 | M | 54 | A | Medium-low | N | T1 | 0.002 |
| 5 | M | 67 | A | Medium-low | Y | T4 | 0.081 |
| 6 | M | 55 | A | Medium | N | T1 | 0.213 |
| 7 | F | 46 | A | Medium | N | T1 | 0.090 |
| 8 | M | 75 | A | Medium-low | Y | T4 | 0.006 |
| 9 | M | 51 | A | Medium-low | Y | T1 | 0.862 |
| 10 | F | 54 | A | Medium-low | Y | T4 | 0.169 |
| 11 | F | 63 | A | Medium-low | Y | T4 | 0.046 |
| 12 | F | 68 | A | Medium | Y | T4 | 0.147 |
| 13 | F | 59 | A | Medium | N | T2 | 1.576 |
| 14 | M | 58 | A | low | Y | T1 | 0.100 |
| 15 | M | 69 | A | low | Y | T1 | 0.424 |
| 16 | M | 54 | A | High-Medium | N | T1 | 0.236 |
| 17 | F | 48 | A | Medium | N | T2 | 6.303 |
| 18 | F | 73 | A | Medium | Y | T1 | 0.454 |
| 19 | F | 52 | A | Medium | Y | T2 | 0.348 |
| 20 | F | 61 | A | Medium-low | N | T2 | 0.190 |
| 21 | F | 53 | A | Medium-low | N | T1 | 1.334 |
| 22 | F | 75 | A | Medium | N | T1 | 0.025 |
| 23 | F | 64 | A | Medium-low | Y | T1 | 0.960 |
| 24 | M | 76 | A | Medium | N | T2 | 0.406 |
| 25 | M | 54 | A | Medium | Y | T1 | 0.419 |
| 26 | F | 54 | A | Medium-low | Y | T2 | 0.509 |
| 27 | F | 53 | A | Medium-low | Y | T2 | 0.000 |
| 28 | M | 54 | A | Medium | Y | T2 | 38.592 |
| 29 | F | 54 | A | Medium-low | Y | T1 | 0.244 |
| 30 | F | 64 | A | Medium-low | Y | T4 | 0.282 |
| 31 | F | 44 | A | Medium | Y | T4 | 1.149 |
| 32 | M | 47 | A | Medium | Y | T4 | 1.772 |
| 33 | F | 38 | A | Medium-low | N | T1 | 10.602 |
| 34 | M | 63 | A | Medium-low | Y | T4 | 0.200 |
| 35 | F | 68 | A | Medium | N | T4 | 0.214 |
| 36 | F | 50 | A | low | N | T4 | 2.251 |
| 37 | M | 53 | A | Medium-low | N | T4 | 0.850 |
| 38 | F | 80 | A | Medium-low | N | T1 | 1.962 |
| 39 | F | 47 | A | Medium-low | N | T4 | 0.296 |
| 40 | M | 64 | A | low | N | T4 | 0.019 |
| 41 | M | 67 | A | Medium-low | N | T4 | 0.032 |
| 42 | F | 54 | A | Medium-low | Y | T4 | 0.291 |
| 43 | M | 47 | A | Medium-low | N | T4 | 0.511 |
| 44 | F | 63 | A | Medium | N | T4 | 0.008 |
| 45 | F | 62 | A | Medium-low | N | T2 | 0.442 |
| 46 | M | 59 | A | Medium-low | N | T4 | 0.529 |
| 47 | M | 55 | A | Medium-low | Y | T4 | 5.135 |
| 48 | M | 60 | A | Medium-low | N | T4 | 0.398 |
| 49 | F | 59 | A | Medium-low | Y | T2 | 0.003 |
| 50 | M | 73 | S | Medium | Y | T2 | 0.043 |
| 51 | M | 61 | S | Medium | Y | T1 | 0.233 |
| 52 | M | 62 | S | Medium-low | Y | T1 | 0.856 |
| 53 | M | 50 | S | low | Y | T1 | 0.001 |
| 54 | M | 79 | S | low | N | T1 | 13.132 |
| 55 | M | 62 | S | Medium-low | Y | T1 | 30.076 |
| 56 | M | 80 | S | low | N | T1 | 0.118 |
| 57 | M | 63 | S | Medium-low | Y | T1 | 2.331 |
| 58 | M | 68 | S | Medium-low | Y | T1 | 0.511 |
| 59 | M | 61 | S | low | N | T1 | 1.076 |

Note: F, female. M, male. Y, Yes. N, No. A, adenocarcinoma. S, squamous cell carcinoma

**Supplemental Table 2**

Table S2. The list of qRT-PCR primers

| Gene | Sequence (5'-3') |
| --- | --- |
| SELENBP1-F | TCCCCAGTATTGCCAGGTCAT |
| SELENBP1-R | CGACTTGGTGCTATCACCGAA |
| GPX1-F | CAGTCGGTGTATGCCTTCTCG |
| GPX1-R | GAGGGACGCCACATTCTCG |
| β-actin-F | CTTAGTTGCGTTACACCCTTTCTTG |
| β-actin-R | ACTGCTGTCACCTTCACCGTTC |

**Supplemental Figure 1**

**
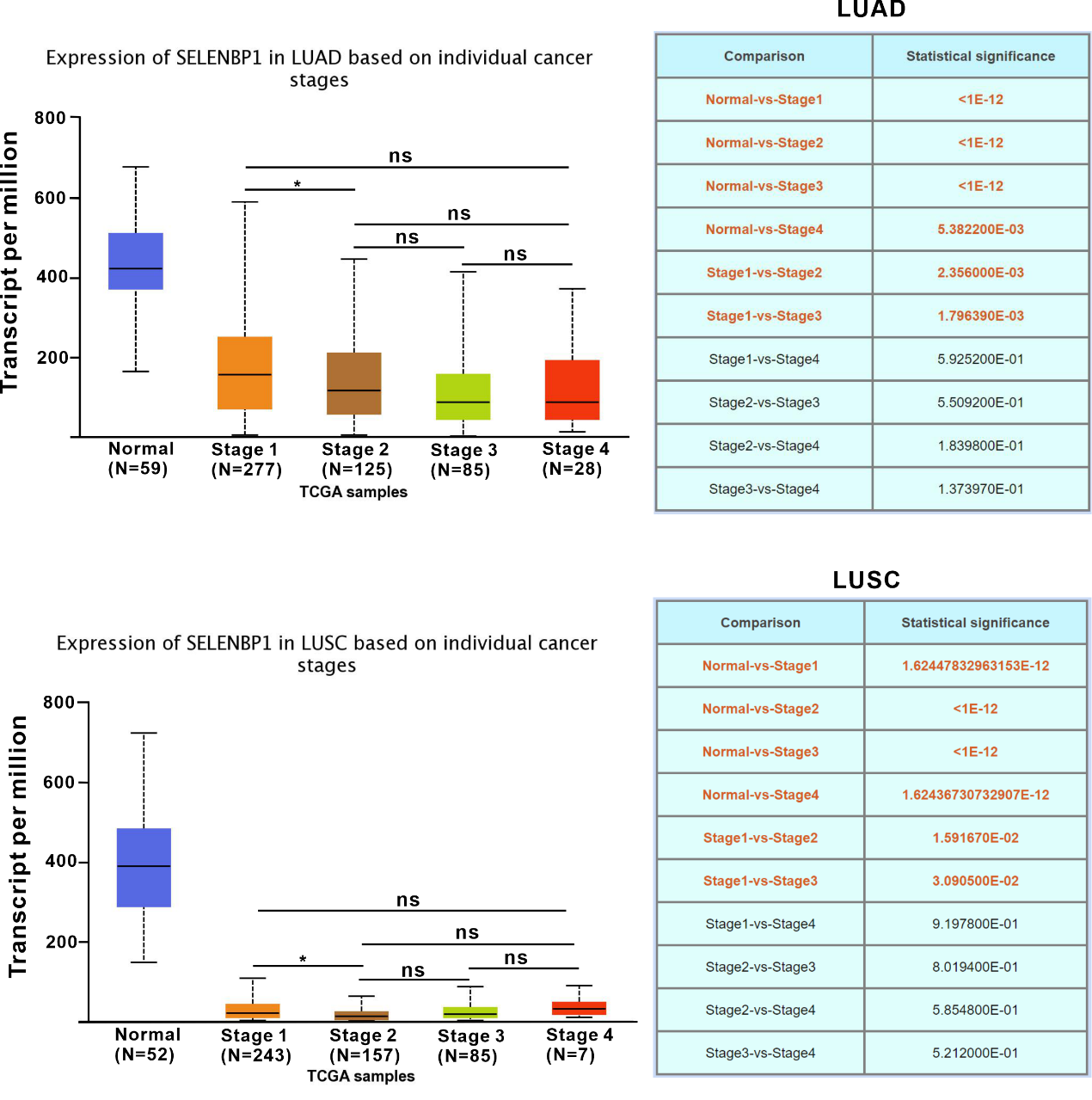
**

**Figure S1. The relationship between the expression of SELENBP1 and the disease stages of NSCLC in TCGA database.** The expression of SELENBP1 has no significance correlation between stage 1 and stage 4, stage 2 and stage 3, stage 2 and stage 4, stage 3 and stage 4 in both LUAD and LUSC, respectively. “*” *P* < 0.05, “ns”, no significant.

**Supplemental Table 3**

Table S3. The relative expression of SELENBP1 in 59 clinical NSCLC tissues

| SELENBP1 Relative Expression | Cases |
| --- | --- |
| Downregulated | 45（76.27%） |
| Upregulated | 13（22.03%） |
| no significance | 1（1.69%） |
| Total | 59（100%） |

**Supplemental Table 4**

Table S4. The IHC score criteria and outcomes of SELENBP1

| Degree | SELENBP1 | | Score | |
| --- | --- | --- | --- | --- |
| Blue | Negative （-） | | 0 | |
| Pale yellow | Weakly positive （+） | | 1 | |
| Brownish yellow | Moderately positive （++） | | 2 | |
| Dark brown | Strongly positive （+++） | | 3 | |
| No. | SELENBP1 IHC staining | | | |
|  | Adjacent non-tumor | Score | Tumor | Score |
| 1 | ＋＋＋ | 3 | ＋ | 1 |
| 2 | ＋＋＋ | 3 | ＋ | 1 |
| 3 | ＋＋＋ | 3 | ＋ | 1 |
| 4 | ＋＋＋ | 3 | － | 0 |
| 5 | ＋＋＋ | 3 | ＋＋ | 2 |
| 6 | ＋＋＋ | 3 | ＋＋ | 2 |
| 7 | ＋＋＋ | 3 | ＋ | 1 |
| 8 | ＋＋＋ | 3 | ＋ | 1 |
| 9 | ＋＋＋ | 3 | ＋ | 1 |
| 10 | ＋＋ | 2 | ＋ | 1 |
| 11 | ＋＋＋ | 3 | ＋＋ | 2 |
| 12 | ＋＋＋ | 3 | ＋ | 1 |
| 13 | ＋＋＋ | 3 | ＋ | 1 |
| 14 | ＋＋＋ | 3 | ＋ | 1 |

**Supplemental Figure 2**


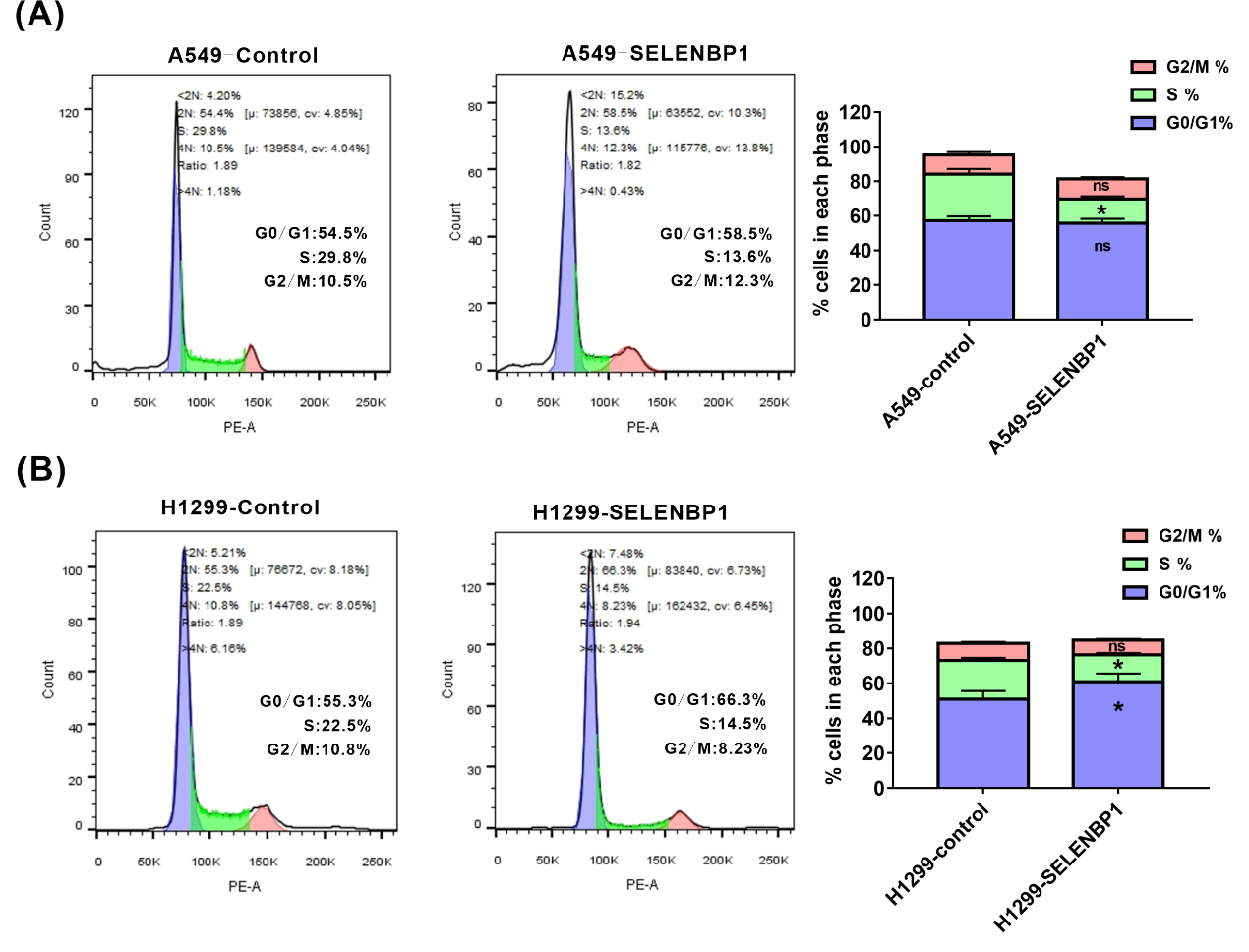


**Figure S2. Overexpression of SELENBP1 inhibited S phase of NSCLC cells.** Flow cytometry analysis was subjected (left), and the ratio of every cell cycle phase (%) (right) in both A549-SELENBP1 cells and control cells (A), H1299-SELENBP1 cells and control cells (B) was calculated. “*” *P* < 0.05, “ns”, no significant, A549-Control group vs. A549-SELENBP1 group, H1299-Control group vs. H1299-SELENBP1 group.

**Supplemental Figure 3**


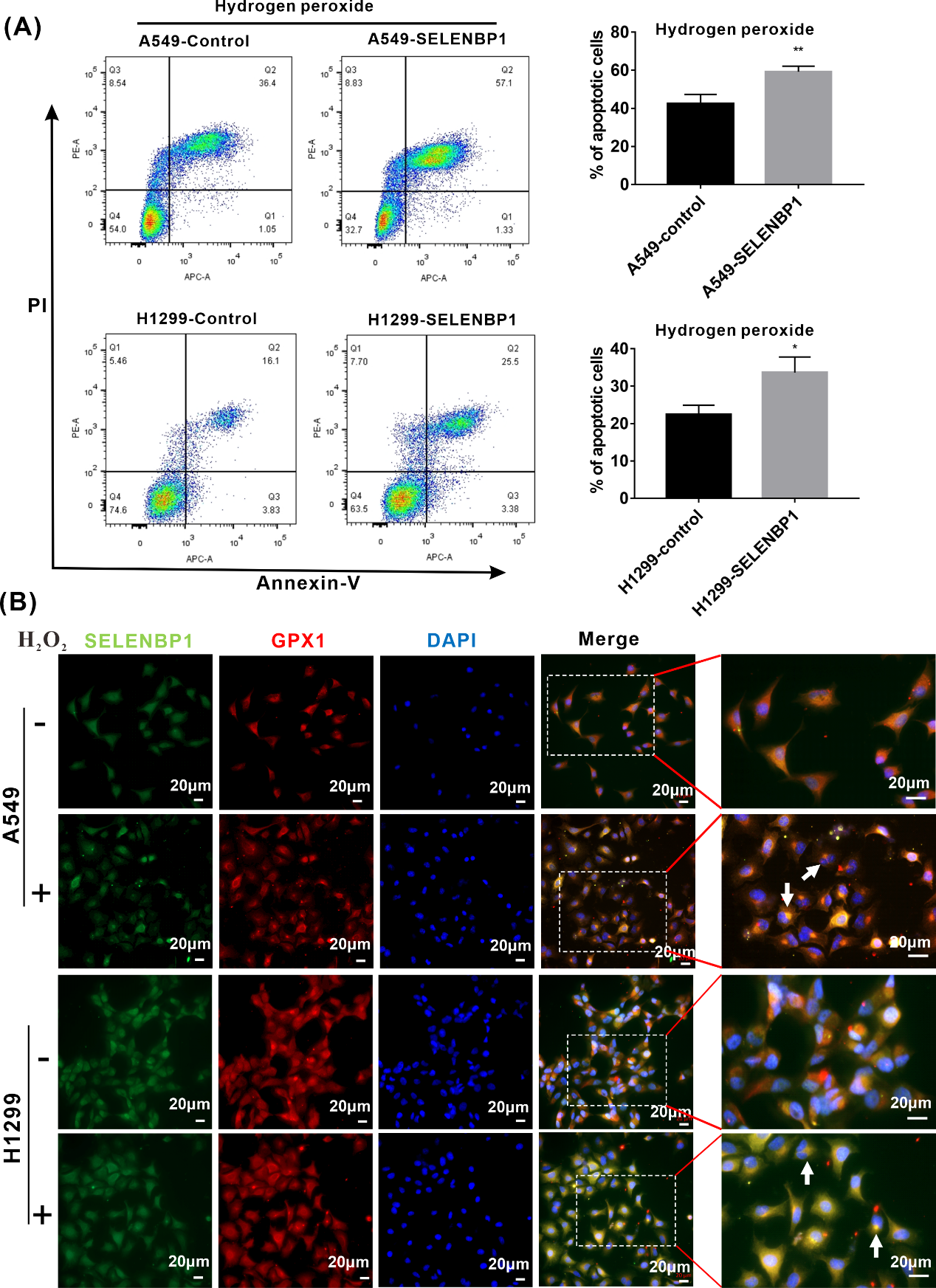


**Figure S3. Overexpression of SELENBP1 inducing the apoptosis of NSCLC cells under high level of oxidative stress might be related to its combining with GPX1 and** **colocalizing in nucleus in vitro.** (A). Flow cytometry analysis was conducted (left), and the ratio of apoptotic cells (%) following hydrogen peroxide treatment for 24h was calculated (N=3) (right), All data were presented as the mean ± SD, unpaired t-test, “*” *P* < 0.05, “**” *P* < 0.01, A549-Control group vs. A549-SELENBP1 group, H1299-Control group vs. H1299-SELENBP1 group. (B) SELENBP1 and GPX1 colocalized in nucleus following hydrogen peroxide treatment for 24h. Staining without primary antibody was used as negative controls. Results were observed under a microscope (×400). Scale bars, 20μm. Images are shown while representative positive stains are indicated by white arrows.

**Supplemental Figure 4**


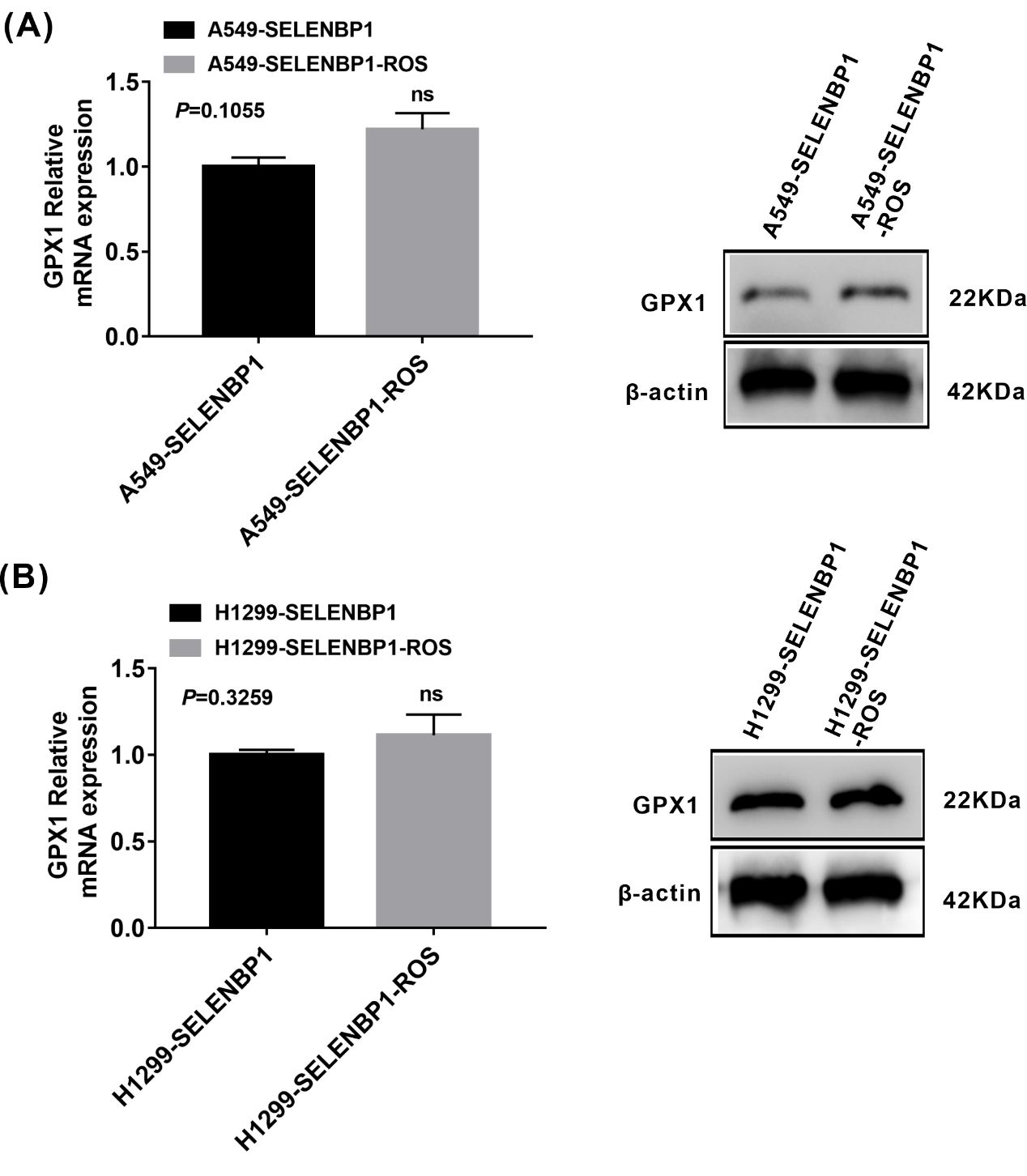


**Figure S4. The mRNA and protein expression of GPX1 did not be affected by overexpression of SELENBP1 under high level of oxidative stress.** The expression of GPX1 in both A549-SELENBP1 cells and control cells (A), H1299-SELENBP1 cells and control cells (B) were measured by qRT-PCR and western blotting under high level of oxidative stress. All data were presented as the mean ± SD, unpaired t-test, “ns”, no significant. A549-SELENBP1 group vs. A549-SELENBP1-ROS group, H1299-SELENBP1 group vs. H1299-SELENBP1-ROS group.
